# Decoding muscle-resident Schwann cell dynamics during neuromuscular junction remodeling

**DOI:** 10.1172/jci.insight.195917

**Published:** 2025-12-23

**Authors:** Steve D. Guzman, Ahmad Abu-Mahfouz, Carol S. Davis, Lloyd P. Ruiz, Peter C.D. Macpherson, Susan V. Brooks

**Affiliations:** 1 Department of Molecular and Integrative Physiology,; 2 Department of Computational Medicine and Bioinformatics, and; 3 Department of Biomedical Engineering, University of Michigan, Ann Arbor, Michigan, USA.

**Keywords:** Cell biology, Muscle biology, Skeletal muscle, Synapses, Transcriptomics

## Abstract

In this study, we used single-cell RNA sequencing to delineate the contributions of muscle-resident Schwann cells to neuromuscular junction (NMJ) remodeling by comparing a model of stable innervation with models of reinnervation following partial or complete denervation. We discovered multiple distinct Schwann cell subtypes, including a terminal Schwann cell subtype integral to the denervation-reinnervation cycle, identified by a transcriptomic signature indicative of cell migration and polarization. The data also characterize 3 myelin Schwann cell subtypes, which are distinguished based on enrichment of genes associated with myelin production, mesenchymal differentiation, or collagen synthesis. Importantly, SPP1 signaling emerged as a pivotal regulator of NMJ dynamics, promoting Schwann cell proliferation and muscle reinnervation across nerve injury models. These findings advance our understanding of NMJ maintenance and regeneration and underscore the therapeutic potential of targeting specific molecular pathways to treat neuromuscular and neurodegenerative disorders.

## Abstract 2

Single-cell RNA sequencing reveals how distinct Schwann cell subtypes and SPP1 signaling coordinate neuromuscular junction repair following partial and complete muscle denervation.

## Introduction

The neuromuscular junction (NMJ) is the terminal synapse of the motor system, orchestrating muscle contraction and voluntary movement. Thus, any degenerative changes at the synapse have the potential to impair contractile function and mobility. The major cellular components of the NMJ are the presynaptic motor neuron, the postsynaptic muscle fiber, and supporting glia. The primary glial cell type at the NMJ is a specialized nonmyelinating Schwann cell (SC) population referred to as terminal SCs (tSCs). tSCs regulate synaptic activity (1) and tSC proliferation is critical for effective muscle reinnervation (2, 3), but the cell biology and physiology of muscle-resident SCs and their response to cues that induce reinnervation remain poorly understood.

To comprehensively assess the role of muscle-resident SCs in NMJ remodeling and muscle fiber reinnervation, we conducted single-cell RNA sequencing (scRNA-Seq) on cells isolated from muscles during recovery from complete denervation postsurgical nerve crush injury and from 2-month-old superoxide dismutase 1–knockout (*Sod1*^–/–^) mice undergoing spontaneous denervation and reinnervation. The *Sod1*^–/–^ mouse model displays widespread and progressive NMJ degeneration and denervation and has been widely used for testing hypotheses focused on the regulators of NMJ structure and function (4–6). A recent revelation from the *Sod1*^–/–^ mouse model is a distinct time window early in life during which known markers of muscle fiber denervation (7–9), acetylcholine receptor (AChR) α (*Chrna*) mRNA levels and mitochondrial reactive oxygen species (mtROS) production, are dramatically but transiently elevated (10). A pattern of increases around 2 months of age and return to baseline by 3 months for both *Chrna* mRNA and mtROS suggests a robust denervation event that is followed by successful reinnervation, suggesting a regenerative window in which NMJ repair processes following spontaneous denervation can be examined.

Here, we rigorously confirmed widespread denervation with successful regeneration between 1 and 3 months of age in *Sod1*^–/–^ mice, supporting the use of young *Sod1*^–/–^ mice as a model of spontaneous recoverable denervation alongside the full denervation associated with surgical nerve crush injury. scRNA-Seq data revealed multiple SC subtypes in muscle, including a tSC subtype that is enriched during the denervation-reinnervation response. This subtype is characterized by a unique transcriptomic signature indicative of cell polarization and morphogenesis. Moreover, we identified multiple myelin-producing SC (mSC) subtypes, 2 of which were enriched with either mesenchymal differentiation genes or collagen-specific genes rather than putative myelin-producing pathways. Across both the spontaneous denervation and surgical nerve crush injury models, secreted phosphoprotein 1 (SPP1) signaling emerged as an important mediator of interactions between mSCs, phagocytic cells, and tSCs, consistently driving SC proliferation associated with successful reinnervation. These findings underscore the universal importance of SPP1 signaling in NMJ dynamics and offer valuable insights into the biology of muscle-resident glial cells, paving the way for innovative therapeutic strategies in denervating conditions like amyotrophic lateral sclerosis (ALS) and age-associated skeletal muscle wasting.

## Results

### scRNA-Seq captures muscle-resident SCs across healthy and denervated muscles.

**Figure 1:**
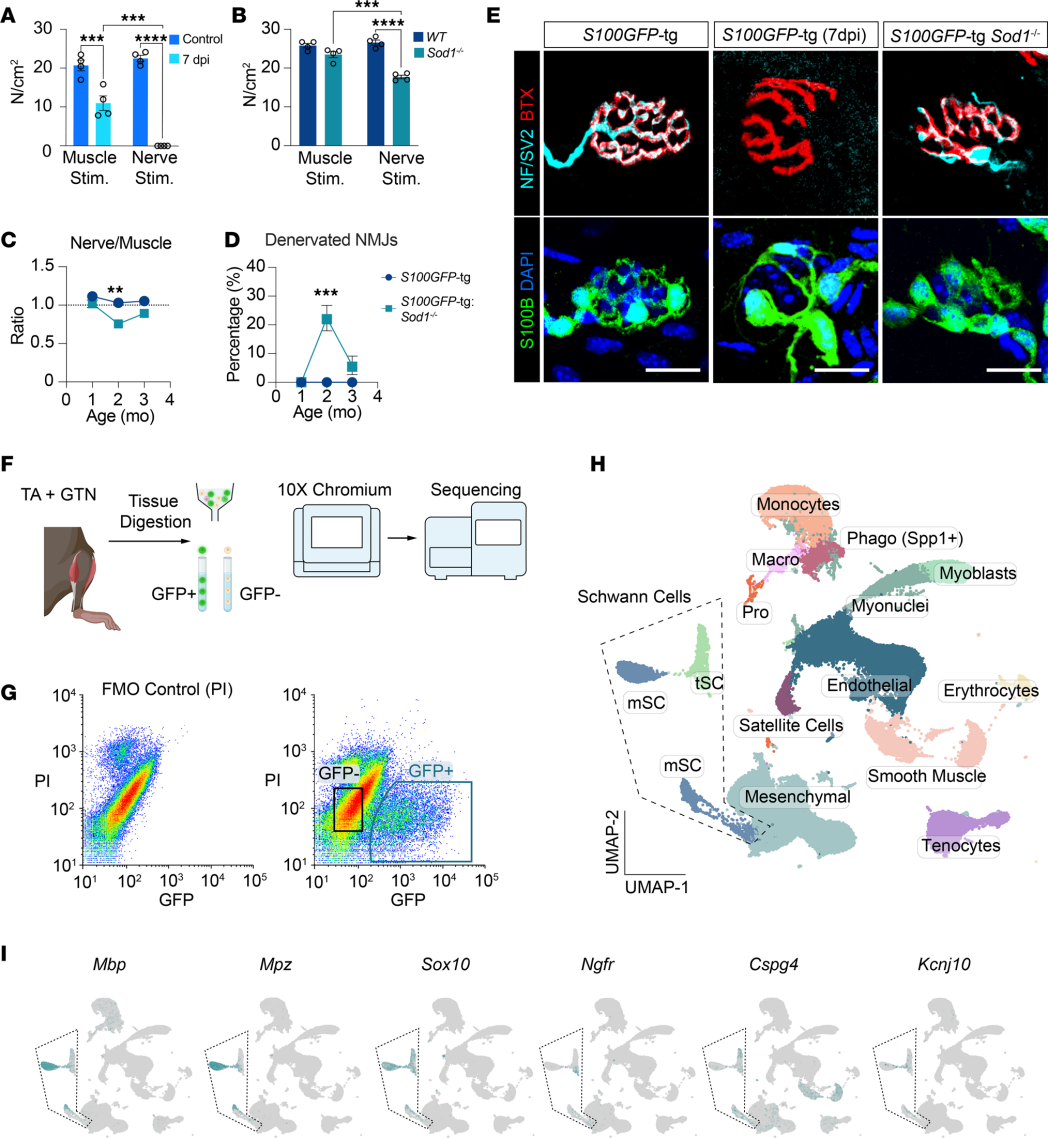
Characterization of muscle denervation in WT and *Sod1*^–/–^ mice and muscle-resident Schwann cell subtypes across different denervation states via scRNA-Seq. (**A** and **B**) Maximum tetanic contraction forces (N/cm^2^) generated by nerve stimulation and direct muscle stimulation in WT mice 7 days post sciatic nerve crush injury (7 dpi) (**A**) and in *S100GFP*-tg *Sod1*^–/–^ mice (**B**), compared to age-matched WT controls (*n* = 4 per genotype). (**C**) Comparison of maximum isometric force ratios elicited by nerve versus direct muscle stimulation across 1–3 months in *S100GFP*-tg *Sod1*^–/–^ (*n* = 3–5) and control mice (*n* = 3–5). (**D**) Percentage of denervated NMJs in gastrocnemius muscles of *S100GFP*-tg and *S100GFP*-tg *Sod1*^–/–^ mice aged 1–3 months. **P < 0.01; ***P < 0.001; ****P < 0.0001 by 2-way ANOVA with Tukey test for multiple testing correction. (**E**) Representative staining of NMJs showing Schwann cells (S100B; green), nerve terminals (NF/SV2; cyan), acetylcholine receptors (AChR; red), and nuclei (DAPI; blue) in 2-month-old *S100GFP*-tg control mice, *S100GFP*-tg mice 7 dpi and *Sod1*^–/–^ mice without nerve injury. Scale bars: 25 μm. (**F**) Experimental workflow: Bilateral gastrocnemius (GTN) and tibialis anterior (TA) muscles were harvested from 2-month-old *S100GFP*-tg, *S100GFP*-tg *Sod1*^–/–^, and *S100GFP*-tg mice 7 dpi, followed by FACS to isolate GFP^+^ and GFP^–^ cells, then processed for scRNA-Seq using the 10X Chromium platform. (**G**) FACS plots showing gating strategies for GFP^+^PI^–^ single cells, with FMO (PI only) controls on the left. (**H**) UMAP plot visualizing 15 distinct cell clusters. (**I**) UMAP plots displaying transcript levels for myelin Schwann cell (mSC) markers (*Mbp*, *Mpz*), general Schwann cell markers (*Sox10*), Schwann cell repair phenotype (*Ngfr*), and terminal Schwann cells (tSC) (*Cspg4*, *Kcnj10*).

SC regenerative responses are activated by axonal injury, but the extent and persistence of the injury can lead to a spectrum of cellular processes within the regenerative timeframe. Utilizing a sciatic nerve crush injury model in SC reporter mice (*S100GFP*-tg), we induced complete muscle denervation, characterized by a total loss of force upon nerve stimulation at 7 days post injury (7 dpi) (Figure 1A). In uninjured 2-month-old *Sod1*^–/–^ mice, the force generated during nerve stimulation was 33% lower than that of *Sod1^WT/WT^* controls, while the force generated in response to direct muscle stimulation did not differ between genotypes (Figure 1B). This finding is indicative of the presence in *Sod1*^–/–^ mice of denervated muscle fibers that were highly contractile but nonresponsive to nerve stimulation. Consistent with published reports of elevated markers of muscle fiber denervation at 2 months of age in *Sod1*^–/–^ mice (10), the neurotransmission impairments we observed in *Sod1*^–/–^ SC reporter mice (*S100GFP*-tg *Sod1*^–/–^) were specifically noted at 2 months of age. The denervation event observed in the muscles 2-month-old *Sod1*^–/–^ mice appears to be transient in nature, as evidenced by the lack of differences in force elicited by nerve and direct muscle stimulation at either 1 or 3 months (Figure 1C). In contrast, there was no functional evidence of denervation in *Sod1^WT/WT^* controls at any time point (Figure 1C). This temporal pattern of innervation loss and subsequent reinnervation in *Sod1*^–/–^ mice correlates with direct assessments of NMJ denervation observed histologically, where the percentage of denervated NMJs was significantly elevated at age 2 months (~23%) and returned to baseline by age 3 months (Figure 1D). Morphological assessments of NMJs in both denervation models and healthy innervated controls confirmed the presence of tSCs across all conditions (Figure 1E). Despite distinct triggering events and varying degrees of denervation between models, tSCs remained a constant feature at NMJs, suggesting underlying transcriptional commonalities among the groups that warranted further investigation.

While the classification of SCs has largely relied on histological examination and scRNA-Seq from nerve extracts (11), transcriptomic data on muscle-resident SCs, especially tSCs, remains scant (12–15). A particular challenge faced by these studies is the rarity of tSCs limiting the cell numbers previously analyzed to as few as 100 individual cells. To overcome this limitation and gain a thorough understanding of the transcriptomic changes in muscle-resident SCs, as well as other cells in the muscle microenvironment during NMJ remodeling, we used scRNA-Seq on pooled tibialis anterior (TA) and gastrocnemius (GTN) muscles from *S100GFP*-tg mice, *S100GFP*-tg *Sod1*^–/–^ mice, and *S100GFP*-tg mice at 7 dpi, all at 2 months of age (Figure 1F). After digesting the muscles, we sorted GFP^+^PI^–^ and GFP^–^PI^–^ single cells using fluorescence-activated cell sorting (FACS) (Figure 1G) and then analyzed the cells by droplet-based scRNA-Seq. Our unbiased clustering approach using uniform manifold approximation and projection (UMAP) classified 54,273 cells (*S100GFP*-tg [*n* = 5,330]; *S100GFP*-tg *Sod1*^–/–^ [*n* = 9,577]; *S100GFP*-tg 7 dpi [*n* = 39,366]) into 12 non-SC clusters and 2 SC clusters, including mSC and tSC clusters (Figure 1H). Both SC clusters (Supplemental Figure 1A; supplemental material available online with this article; https://doi.org/10.1172/jci.insight.195917DS1) displayed classic SC markers such as *S100b* and *Sox10* (Figure 1I and Supplemental Figure 1A) and no SC clusters were identified in GFP^–^ cells. Notably, clusters of mSCs were marked by expression of myelin-associated genes like *Mbp* and *Mpz* (Figure 1I), while the tSC cluster presented expression of recently reported non-mSC markers, *Cspg4* (*Ng2*) and *Kcnj10* (*Kir4.1*) (Figure 1I) (12, 14). Additionally, the tSC cluster prominently expressed *Ngfr* (p75NTR), a classical SC repair marker. *S100GFP*-tg and *S100GFP*-tg *Sod1*^–/–^ samples displayed a comparable total number of SCs (1,649 and 2,101, respectively); however, relatively few SCs (*n* = 369) were isolated from *S100GFP*-tg 7-dpi mice, despite having nearly 4-fold more total cells than the other groups. These muscles (7 dpi), however, were highly enriched for mesenchymal cells (+30%), monocytes (+10), and a *Spp1^+^Cora1a^+^* cell cluster we termed as phagocytic (Supplemental Figure 1B). Interestingly, we found many GFP^+^ non-SC clusters by FACS that expressed *Gfp* mRNA but had little to no expression of *S100b* (Supplemental Figure 2). These cells were abundant in *S100GFP*-tg 7-dpi mice, and likely suggestive that SC dedifferentiation was underway and prevalent at this injury time point, but this remains to be experimentally validated.

### Differential SC dynamics during NMJ remodeling.

**Figure 2:**
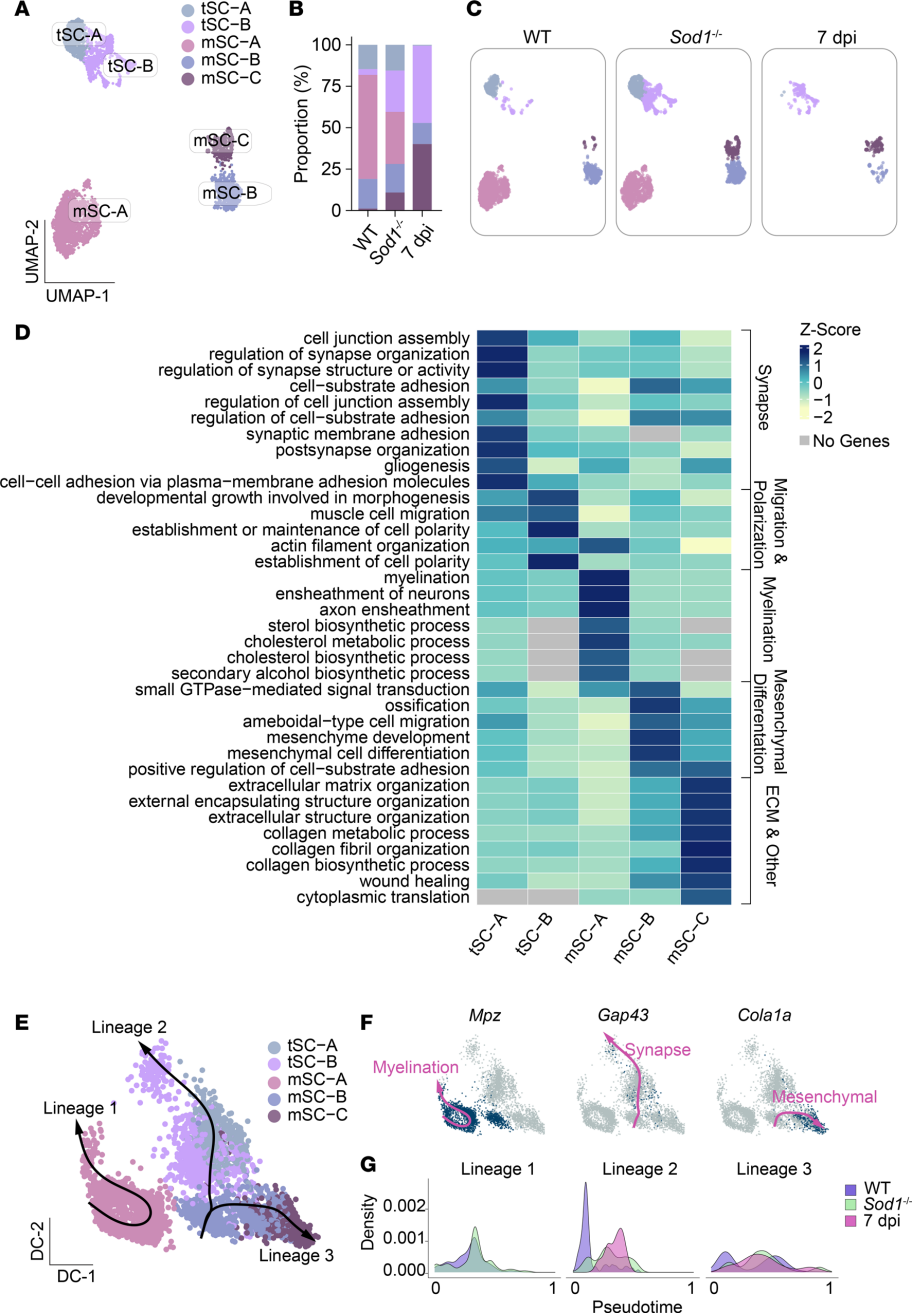
Dynamics of muscle-resident Schwann cell subtypes in healthy and remodeling neuromuscular junctions. (**A**) UMAP visualization of reclustered muscle-resident Schwann cell clusters, identifying 2 terminal Schwann cell subclusters (tSC-A and tSC-B) and 3 myelin Schwann cell subclusters (mSC-A, mSC-B, mSC-C). (**B**) Proportions (%) of each Schwann cell subcluster, with corresponding UMAP plots (**C**) across conditions in WT, *Sod1^–/–^*, and 7-dpi mice. (**D**) Heatmap illustrating gene ontology (GO) pathway analysis results, with enriched biological processes for each Schwann cell cluster presented as *z* scores of normalized –log(*P* value) for each GO term. (**E**) Diffusion map showing Schwann cell subclusters with 3 trajectory lineages from slingshot analysis superimposed. (**F**) UMAP feature plots indicating expression patterns of key genes (*Mpz*, *Gap43*, *Cola1a*), highlighting their distribution within lineages 1, 2, and 3, respectively. (**G**) Variation in cell density over pseudotime for each lineage in WT, *Sod1*^–/–^, and 7-dpi mice, reflecting differential engagement in nerve repair processes.

Reclustering of SC transcriptomes revealed 5 subtypes, including 3 mSCs (mSC-A, mSC-B, mSC-C) and 2 distinct tSC subgroups, termed tSC-A and tSC-B (Figure 2, A–C). Notably, there was a significant increase in the tSC-B subgroup under both denervation conditions, representing approximately 20% of the SCs in 2-month-old *S100GFP*-tg *Sod1*^–/–^ mice and approximately 50% in the *S100GFP*-tg 7-dpi mice but only 4% in the *S100GFP*-tg controls. Also common to both denervation models was a dramatic reduction in the proportion of mSC-A, representing 63% of the SCs in *S100GFP*-tg, but only 31% in *S100GFP*-tg *Sod1*^–/–^, and not detected in *S100GFP*-tg 7-dpi mice, as was also true for tSC-A. Additionally, the mSC-B and mSC-C clusters were more prevalent in the denervation models than in controls.

Pathway analysis highlighted that the tSC-A cluster was associated with synapse organization and structural pathways (Figure 2D and Supplemental Figure 3A), while tSC-B showed enrichment for biological processes tied to migration, cell polarization, and morphogenesis (Figure 2D and Supplemental Figure 3B). The mSC-A cluster primarily engaged classical myelin production pathways, including axon ensheathment, myelination, and cholesterol metabolism (Figure 2D and Supplemental Figure 3C). Interestingly, our data suggest that mSC-B is characterized by gene expression programs defined by mesenchymal differentiation (Figure 2D and Supplemental Figure 3D), consistent with findings from studies of peripheral nerve injury in which SCs are reported to express epithelial-mesenchymal transition (EMT) markers and respond to transforming growth factor β (TGF-β) signaling, a well-known inducer of EMT genes (16, 17). Finally, mSC-C was linked with extracellular matrix (ECM) organization and collagen production (Figure 2D and Supplemental Figure 3E).

To better understand the dynamic response of SC subpopulations during NMJ remodeling, we performed Slingshot trajectory analysis (18) on a diffusion map–reduced space of muscle-resident SCs (Figure 2E). This analysis revealed 3 primary lineages. Lineage 1, which originates in the mSC-A cluster, demonstrated enrichment for myelin-associated genes such as *Mpz* (Supplemental Figure 3C), underscoring its involvement in myelination and axonal support (Figure 2, E and F). The other 2 lineages arise from the mSC-B cluster and coursed through nonmyelinating tSC subpopulations. Lineage 2 was characterized by an enrichment of synapse-related genes, such as *Gap43* (Figure 2E and Supplemental Figure 3A), highlighting its role in synaptic maintenance and regeneration. Meanwhile, Lineage 3 extended toward the mSC-C cluster, which showed enrichment for ECM and mesenchymal differentiation genes, including *Col1a1*, suggesting its involvement in structural remodeling and repair (Figure 2, E and F, and Supplemental Figure 3E).

Our analysis across different experimental groups revealed a complete absence of myelination-associated Lineage 1 SCs in WT (*S100GFP*-tg) mice at 7 dpi. This finding aligns with established knowledge that acute nerve injury not only suppresses myelination-specific pathways but also leads to the reduction of SCs contributing to these pathways, while concurrently activating myelin clearance, a typical immediate response to nerve damage (19) (Figure 2G). Conversely, control noninjured *S100GFP*-tg mice progressed more rapidly along the myelination trajectory, indicating robust myelination activity from the onset of the predicted trajectory. *Sod1*^–/–^ mice–derived muscle-resident SCs, representing a model of spontaneous noninjurious denervation of a fraction of the fibers within the muscle, displayed a moderated progression in this lineage. Lineage 2, associated with synaptic maintenance and regeneration, revealed increasing cell density with pseudotime, beginning with the *S100GFP*-tg controls and progressively increasing in *S100GFP*-tg *Sod1*^–/–^ and 7-dpi SCs. The pattern of increasing cell density in Lineage 2 for both *S100GFP*-tg *Sod1*^–/–^ and 7-dpi SCs is consistent with increased demands for synaptic remodeling in response to denervation through the remodeling trajectory. Lastly, Lineage 3, linked to ECM remodeling and mesenchymal differentiation, showed pronounced enrichment in the 7-dpi SCs, indicative of a shift toward repair functions following acute denervation. Both *S100GFP*-tg and *Sod1*^–/–^ SCs exhibited phases of activation within this lineage, indicating ongoing remodeling activities during neuromuscular adaptation and repair (Figure 2G).

### The remodeling of NMJs is associated with increased numbers of tSCs and larger synaptic areas.

**Figure 3:**
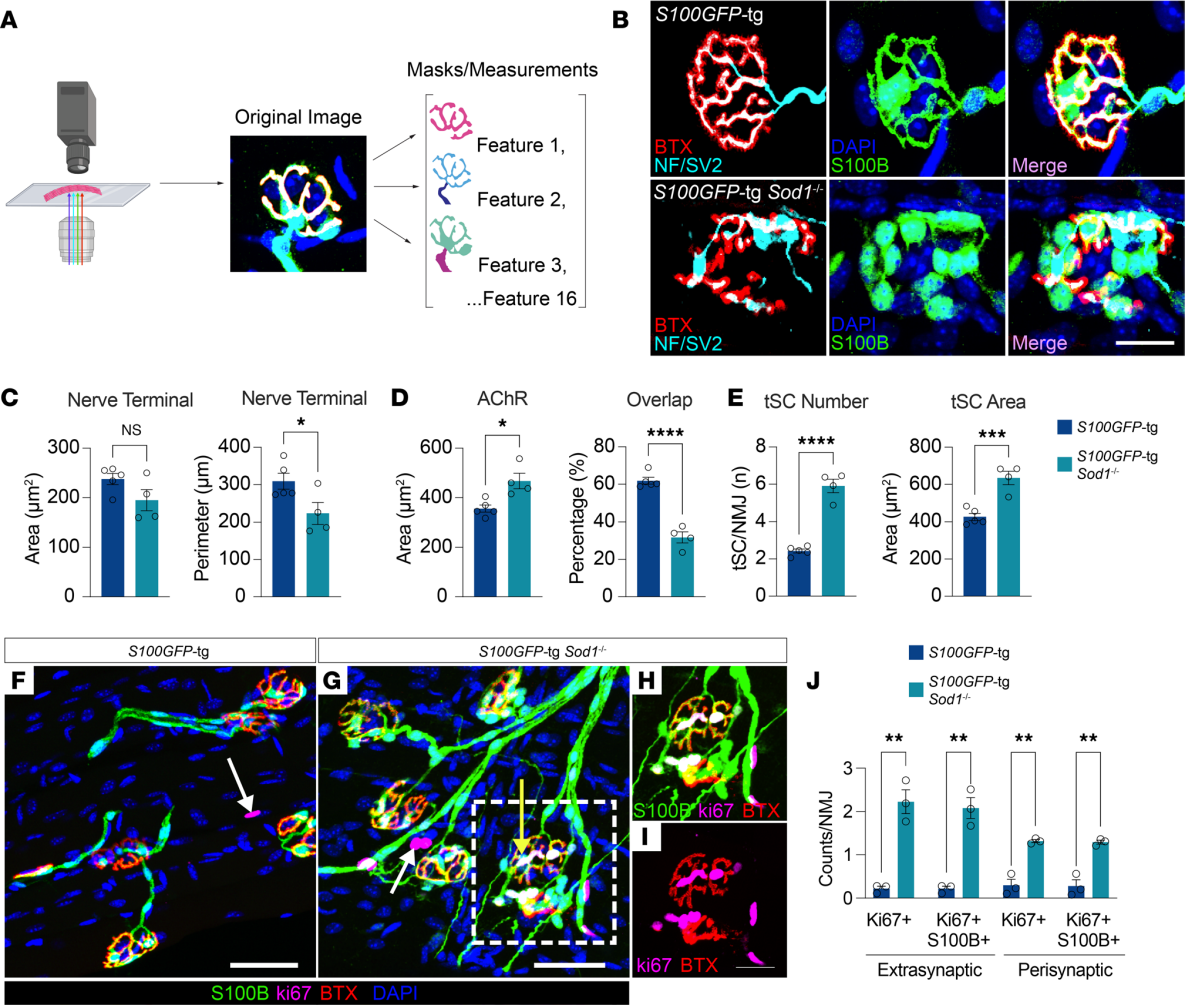
The remodeling of neuromuscular junctions (NMJs) is associated with greater tSC numbers, larger synaptic areas, and enhanced proliferation in *S100GFP*-tg *Sod1*^–/–^ mice. (**A**) Schematic of NMJ analysis, consisting of collecting NMJs images from muscle fiber bundles followed by the generation of feature masks and their measurements. Additional details on the generation of the masks are provided in Supplemental Figures 3 and 4 and Methods. (**B**) NMJs stained for S100B (Schwann cells; green), NF/SV2 (nerve; cyan), AChRs (α-bungarotoxin, BTX; red), and nuclei (DAPI; blue) in 2-month-old *S100GFP*-tg control and *S100GFP*-tg *Sod1*^–/–^ mice. (**C**–**E**) Quantification of nerve terminal area, nerve terminal perimeter, AChR area, percentage overlap between AChR area and nerve terminal area, and tSC number and tSC area. Muscle fiber imaging from *S100GFP*-tg (**F**) and *S100GFP*-tg *Sod1*^–/–^ (**G**) mice, immunostained for S100B (green), Ki67 (magenta), AChR (red), and nuclei (blue). White arrows point to extrasynaptic nuclei positive for Ki67 but lacking endogenously expressed GFP and or S100B immunostaining, while the yellow arrow highlights a perisynaptic Ki67^+^GFP^+^ nucleus. (**H**) Enlarged view of 2 NMJs from the highlighted region in **G**, detailing the S100B, Ki67, and BTX stains. (**I**) The same NMJs from **H**, but focused on BTX and Ki67, revealing multiple Ki67^+^ nuclei in close proximity to the endplate. (**J**) Quantification of extrasynaptic and perisynaptic nuclei either singly labeled for Ki67 or double labeled for Ki67 and GFP. Values for all features across all NMJs analyzed are provided in Supplemental Figure 4. Open circles indicate average for each individual mouse of no fewer than 20 NMJs analyzed per muscle and bars represent means across animals ± SEM. *P < 0.05; **P < 0.01; ***P < 0.001; ****P < 0.0001 by 2-tailed unpaired *t* test (**C**–**E**, vs. *S100GFP***-tg** *Sod1***^–/–^**) and (**J**). Scale bars: 25 μm (**B**) and 50 μm (**F** and **G**). In **C**–**E**, *n* = 4–5/group; **J**, *n* = 3/group.

Given the substantial capture of muscle-resident SCs in 2-month-old *Sod1*^–/–^ mice compared with the 7-dpi mice, and the recognition of this period as a crucial phase for SC-mediated repair against denervation, we undertook a focused examination of cellular and morphological changes during the denervation-reinnervation cycle in *Sod1*^–/–^ mice. We performed detailed imaging analyses on fixed fiber bundles from *S100GFP*-tg *Sod1*^–/–^ and *S100GFP*-tg control mice at 1, 2, and 3 months of age (Figure 3, A and B). Our comprehensive analysis defined NMJ structure, accounting for 16 distinct features related to pre- and postsynaptic structures, including tSC number and morphology, allowing us to track the dynamics of NMJ remodeling and SC activity over a transient repair period. Detailed descriptions of each morphological feature appear in Supplemental Figure 4. In muscles from 2-month-old mice, our findings revealed overall smaller values for *S100GFP*-tg *Sod1*^–/–^ mice compared with fully innervated controls in nerve terminal perimeter and overlap of the nerve terminal with AChRs by 33% and 60%, respectively (Figure 3, C and D). Meanwhile, the area of AChRs was observed to be 30% larger in *S100GFP*-tg *Sod1*^–/–^ mice compared with controls, and the total tSC area and number of tSCs per NMJ were 60% and 3-fold greater, respectively, in the *S100GFP*-tg *Sod1*^–/–^ group (Figure 3E). As a result of the large increase in tSC number, the terminal area per tSC was 38% smaller in *S100GFP*-tg *Sod1*^–/–^ mice. Given that both central and peripheral glia respond to neuronal injury by increasing cell number (2, 20), increased tSC number and area at the NMJ are likely acting to protect or promote NMJ integrity during the early neuromuscular events linked to the *Sod1*^–/–^ NMJ remodeling phenotype.

Both the degree of overlap and synaptic area serve as indicators of the physical congruence of the pre- and postsynaptic components (21). These markers are robust histological indicators of nerve-to-muscle connections. In *S100GFP*-tg *Sod1*^–/–^ mice, the overlap between the nerve terminal and AChRs was markedly decreased at 2 months compared with controls; however, by 3 months, this overlap aligned with that of the *S100GFP*-tg controls (Supplemental Figure 5). Reestablishment of control levels of overlap could be explained by presynaptic responses, compensatory postsynaptic changes, or a combination. The present analysis revealed an expansion in synaptic area between 2 and 3 months in *S100GFP*-tg *Sod1*^–/–^ mice driven largely by an enlargement in nerve terminal area at 3 months (Supplemental Figure 4). Collectively, the restoration at 3 months in the degree of overlap coupled with the reduction in denervated NMJs (characterized by NMJs with less than 10% overlap) (Figure 1D) strongly support reinnervation.

Based on our findings that NMJs in 2-month-old *Sod1*^–/–^ mice were characterized by higher numbers of tSCs than observed in the other groups, we aimed to verify proliferation of S100GFP^+^ cells at the NMJs in these mice. To directly test this, we stained muscle bundles with an anti-Ki67 antibody (Figure 3, F–I) and found numerous Ki67^+^ nuclei in *Sod1*^–/–^ mice compared with age-matched WT mice. Moreover, the majority of Ki67^+^ nuclei originated from cells that were also S100GFP^+^. Ki67^+^ cells were observed both in perisynaptic (overlapping AChR staining) and extrasynaptic locations (within 50 μm of the nearest NMJ) (Figure 3J). These observations corroborate our earlier finding of a 3-fold increase in tSCs in *Sod1*^–/–^ mice at 2 months of age when compared with WT controls (Figure 3E).

Our functional and morphological data strongly support the existence of a key regenerative window centered around 2 months of age in *Sod1*^–/–^ mice that is ideal for studying the cellular and molecular changes in muscle SCs during NMJ remodeling and reinnervation. We also present compelling evidence that this remodeling process includes, at least in part, proliferation of SCs located in the muscle at the NMJ and a promotion of increased synaptic area through nerve terminal growth. Thus, we next aimed to define key intercellular signals that regulate SC dynamics during the remodeling of the NMJ.

### Intercellular communication network analysis reveals an SPP1 signaling dynamic between mSCs and tSCs.

**Figure 4:**
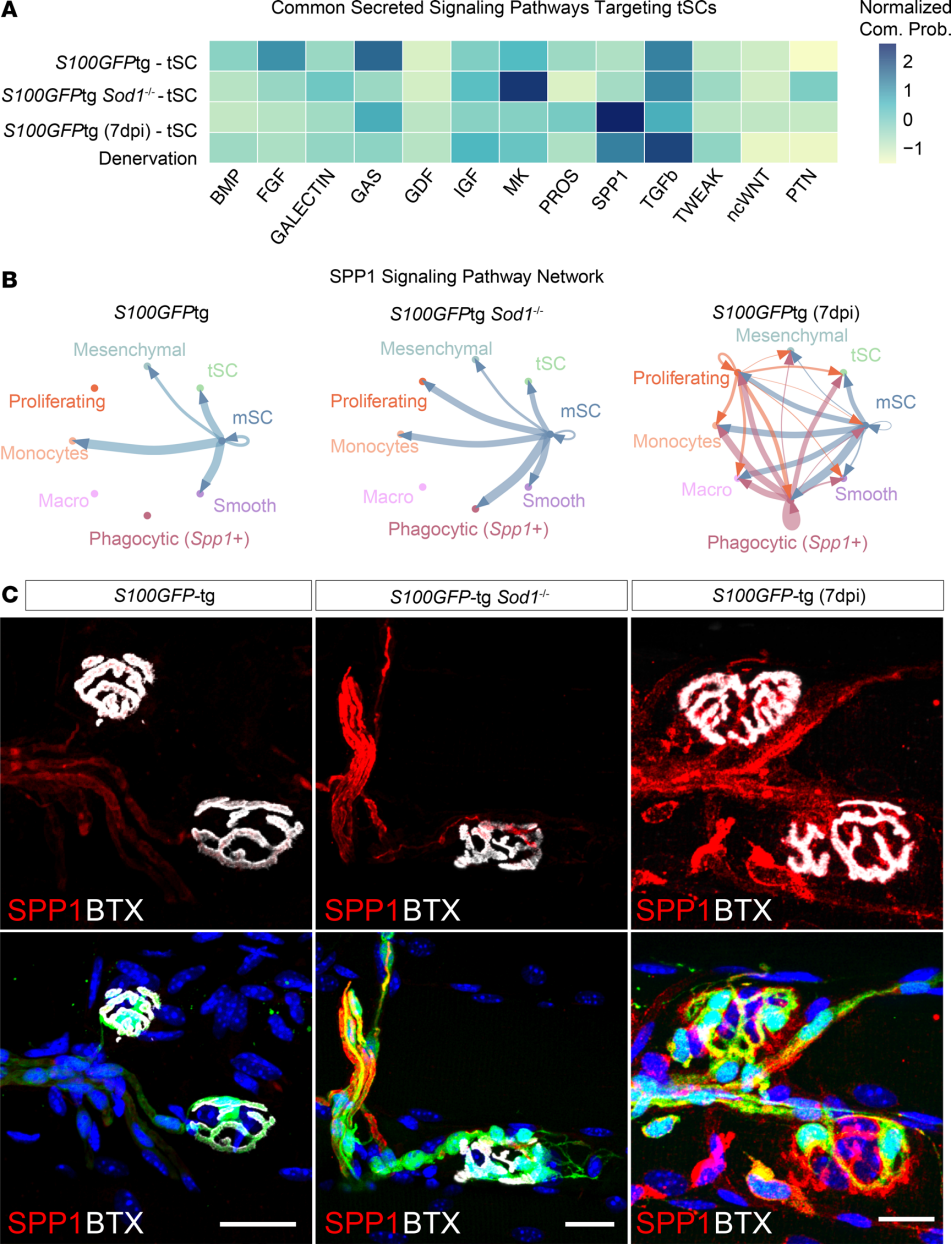
Intercellular communication suggests an SPP1 signaling dynamic between mSCs and tSCs. (**A**) Heatmap showing the common significantly enriched secretion signaling pathways across WT, *Sod1*^–/–^, 7 dpi, and Denervation (combined *Sod1*^–/–^ + WT 7 dpi) targeting tSCs. Coloring of the heatmap is based on the normalized (*z* score) communication probabilities. (**B**) Circle plots displaying the SPP1 signaling network across cell clusters for *S100GFP*-tg, *S100GFP*-tg *Sod1*^–/–^, and *S100GFP*-tg (7 dpi) mice. The thickness of connecting lines represents the communication likelihood between paired cell clusters, with arrowheads demarcating communication directionality. (**C**) Representative immunofluorescence images of NMJs stained for SPP1 (red), GFP (green), AChRs (BTX, white), and nuclei (DAPI, blue). Scale bars: 20 μm.

Based on the unique location of tSCs at the NMJ, signaling to these cells is key to understanding the dynamics of this specialized environment. To explore how tSCs interact with other cellular components within the niche during NMJ remodeling, we conducted an intercellular communication network analysis employing CellChat (22). Utilizing our scRNA-Seq data from tSCs, mSCs, mesenchymal progenitors, macrophages, and smooth muscle cells we identified shared secreted signaling pathways that target tSCs in all experimental groups, including a combined denervation group encompassing both *S100GFP*-tg *Sod1*^–/–^ and *S100GFP*-tg 7-dpi models (Figure 4A). TGF-β and SPP1 signaling emerged as the pathways with the highest normalized communication probabilities in the denervation group, implying important roles in NMJ remodeling in both spontaneous and injurious denervation conditions.

Our finding of marked induction of SPP1 signaling in our denervation groups is compelling in light of a recent study finding that SPP1 promotes SC proliferation and survival, and its expression is notably upregulated in mSCs following human peripheral nerve injury (23). Exploring SPP1 signaling in our dataset, we identified a predominant expression of *Spp1* in mSCs (Figure 4B). While SPP1 appears to communicate with several cell types in *S100GFP*-tg mice, a greater number of cell type receivers was inferred in *Sod1*^–/–^ mice (5 vs. 7) (Figure 4B), which included the addition of proliferating and phagocytic (*Spp1*^+^) cells. Data from *S100GFP*-tg 7-dpi mice also revealed inferred SPP1 signaling originated from phagocytic (*Spp1*^+^), mSCs, and proliferating cell clusters, while the phagocytic (*Spp1*^+^) cell cluster in *Sod1*^–/–^ mice was not predicted to communicate via SPP1 with other cells, likely owing to the limited number of these cells identified in these mice. The SPP1 signaling was primarily predicted to act through *Cd44*, and various integrin dimer combinations with *Itgav*, *Itgb1*, *Itgb3*, and *Itgb5* (Supplemental Figure 6A).

To validate our CellChat findings, we performed targeted RT-qPCR to evaluate the expression levels of key genes within the SPP1 pathway in GTN muscles isolated from 2-month-old *S100GFP*-tg and *S100GFP*-tg *Sod1*^–/–^ mice (Supplemental Figure 6B). We showed upregulation of *Tgfb1*, *Tgfbr2*, and *Spp1* in *Sod1*^–/–^ mice. While *Cd44* levels remained unchanged, we detected a borderline significant elevation in *Cd44v6* (*P* = 0.052), the specific receptor variant of CD44 known to bind SPP1. We next sought to confirm our scRNA-Seq and bioinformatic findings by pinpointing the protein localization of SPP1 in muscle fibers. Histological analysis using an anti-SPP1 antibody on fixed muscle fiber bundles revealed pronounced localization of SPP1 associated with GFP^+^ SCs near the NMJ, with intensified staining observed in *Sod1*^–/–^ mice compared with controls (Figure 4C). In contrast to muscles from control and 2-month-old *Sod1*^–/–^ mice, in *S100GFP*-tg 7-dpi muscles, SPP1 protein localization was observed in both GFP^+^ cells and GFP^–^ cells. It seems likely that the GFP^–^ cells represent the *Spp1*^+^ cell clusters in our scRNA-Seq dataset. Immunostaining also confirmed the localization of CD44 protein at the NMJ and within S100B^+^ SCs (Supplemental Figure 6C). Taken together, our findings illuminate SPP1 cell signaling as a pathway involving mSCs, phagocytic (SPP1^+^) cells, and tSCs, which could play a role in enhancing the tSC proliferation and survival important for successful NMJ remodeling and reinnervation.

### SPP1 gene expression is markedly increased in muscles following nerve injury.

**Figure 5:**
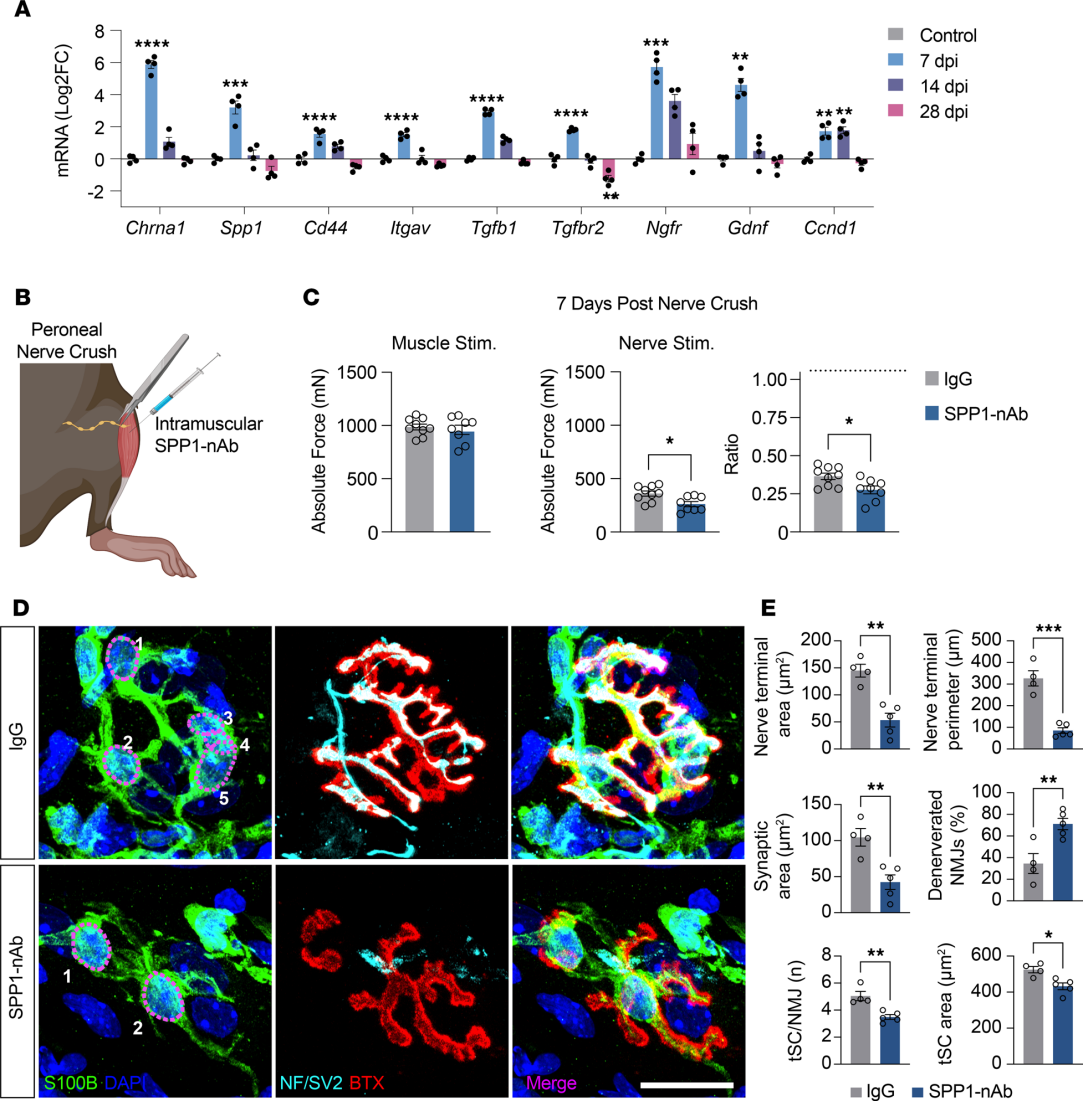
SPP1 signaling promotes tSC proliferation and muscle fiber reinnervation after nerve injury. (**A**) Following sciatic nerve crush injuries, gastrocnemius (GTN) muscles from C57BL/6J mice were collected at 0 (control), 7, 14, and 28 days postinjury (dpi). mRNA expression levels of denervation markers (*Chrna1*), components of SPP1 signaling (*Spp1*, *Cd44*, *Itgav*, *Tgfb1*, *Tgfbr2*), genes linked to SC-mediated nerve regeneration (*Ngfr*, *Gdnf*), and cell proliferation (*Ccnd1*) at each time point are presented. (**B**) Peroneal nerve injuries were induced, and tibialis anterior (TA) muscles were intramuscularly injected with either SPP1-nAb or IgG at time of injury and every 2 days thereafter. (**C**) Data are shown for force (mN) evoked by direct muscle stimulation, with nerve stimulation, and ratio of force elicited by nerve and direct muscle stimulation at 7 dpi for IgG- (gray) and SPP1-nAb–treated (blue) groups. (**D**) Representative immunofluorescence images of NMJs at 7 dpi stained for S100B (green), nuclei (DAPI, blue), AChRs (BTX, red), and NF/SV2 (cyan). (**E**) Quantification of NMJ nerve terminal area and perimeter, synaptic area, percentage of denervated (>10% overlap) NMJs, tSC number and area. Open circles indicate values for individual mice and bars represent the mean across animals ± SEM. Scale bars: 25 μm. *P ≤ 0.05, **P ≤ 0.01, ***P ≤ 0.001, ****P ≤ 0.0001 by 2-tailed unpaired *t* test (IgG vs. SPP1-nAb) or 1-way ANOVA with Dunnett’s test; comparison against the single control uninjured group (7, 14, 28 dpi vs. control) for multiple comparisons. In **A**, *n* = 4/group; **C**, *n* = 8/group; **E**, *n* = 4–5/group.

To explore whether an acute recoverable nerve injury alters the gene expression dynamics of SPP1 signaling in skeletal muscle, we performed sciatic nerve crush procedures on 2-month-old mice (Figure 5A) and assessed transcript levels of denervation response pathways and SPP1 signaling in naive noninjured controls, and 7, 14, and 28 dpi. We observed a striking elevation in *Spp1* expression and its receptors *Cd44* and *Itgav* peaking at 7 dpi and subsequently reverting to baseline levels by 14 dpi (Figure 5A). This temporal trend closely parallels the known post-nerve-injury gene expression pattern of *Chrna1* (9), suggesting an inverse relationship with muscle innervation. In addition, *Tgfb1* and its receptor *Tgfbr2*, proposed to be upstream of SPP1 signaling, were also elevated at 7 dpi, with *Tgfbr2* expression being reduced at 28 dpi compared with uninjured controls. *Ngfr* and *Gdnf*, which are associated with SC-mediated nerve regeneration, were also highly expressed at 7 dpi, while the cell proliferation gene *Ccnd1* was elevated at 7 dpi and persisted at high levels out to 14 dpi. The pronounced *Spp1* expression during the initial nerve regeneration phase and its subsequent normalization consistent with known muscle reinnervation milestones implicates the potential involvement of SPP1 in muscle reinnervation.

### Inhibition of muscle SPP1 after acute nerve injury results in reduced muscle reinnervation and fewer tSCs.

To determine the role of SPP1 signaling in muscle reinnervation and its potential mediation of tSC responses following nerve injury, we performed nerve crush injuries on control *S100GFP*-tg mice and administered intramuscular injections of either an SPP1-neutralizing antibody (SPP1-nAb) or a species-matched control IgG (Figure 5B). To expedite the onset of muscle reinnervation and thereby reduce the number of muscle injections, nerve crushes were performed near the nerve entry point to the TA muscle, with injections administered at the time of injury and every 2 days thereafter. At 7 dpi, recovery of functional neurotransmission was evaluated by comparing nerve- and muscle-evoked contractile responses. Direct muscle stimulation revealed no difference in maximal force production between IgG- and SPP1-nAb–treated groups; however, nerve-evoked muscle forces were 34% lower in the SPP1-nAb group (Figure 5C). Likewise, the ratio of nerve- to muscle-evoked force was 29% lower in muscles treated with SPP1-nAb compared with those receiving control IgG, suggesting that neutralization of intramuscular SPP1 impaired or delayed functional reinnervation.

We next assessed tSC morphology and NMJ structure in fixed muscle fiber bundles at 7 dpi (Figure 5E). Muscles treated with SPP1-nAb exhibited fewer tSCs per NMJ and reduced tSC area compared with IgG controls. Additionally, the SPP1-nAb group displayed marked decreases in nerve terminal area, perimeter, and total synaptic area. The proportion of denervated muscle fibers was substantially higher in SPP1-nAb–treated mice relative to IgG controls (71% vs. 34%), and NMJ synaptic area was correspondingly reduced. Together, these findings indicate that SPP1 signaling promotes effective muscle reinnervation and supports tSC expansion at the NMJ following nerve injury.

### Single-cell profiling reveals that SPP1 neutralization stalls tSCs mid-trajectory and blunts late repair programs.

**Figure 6:**
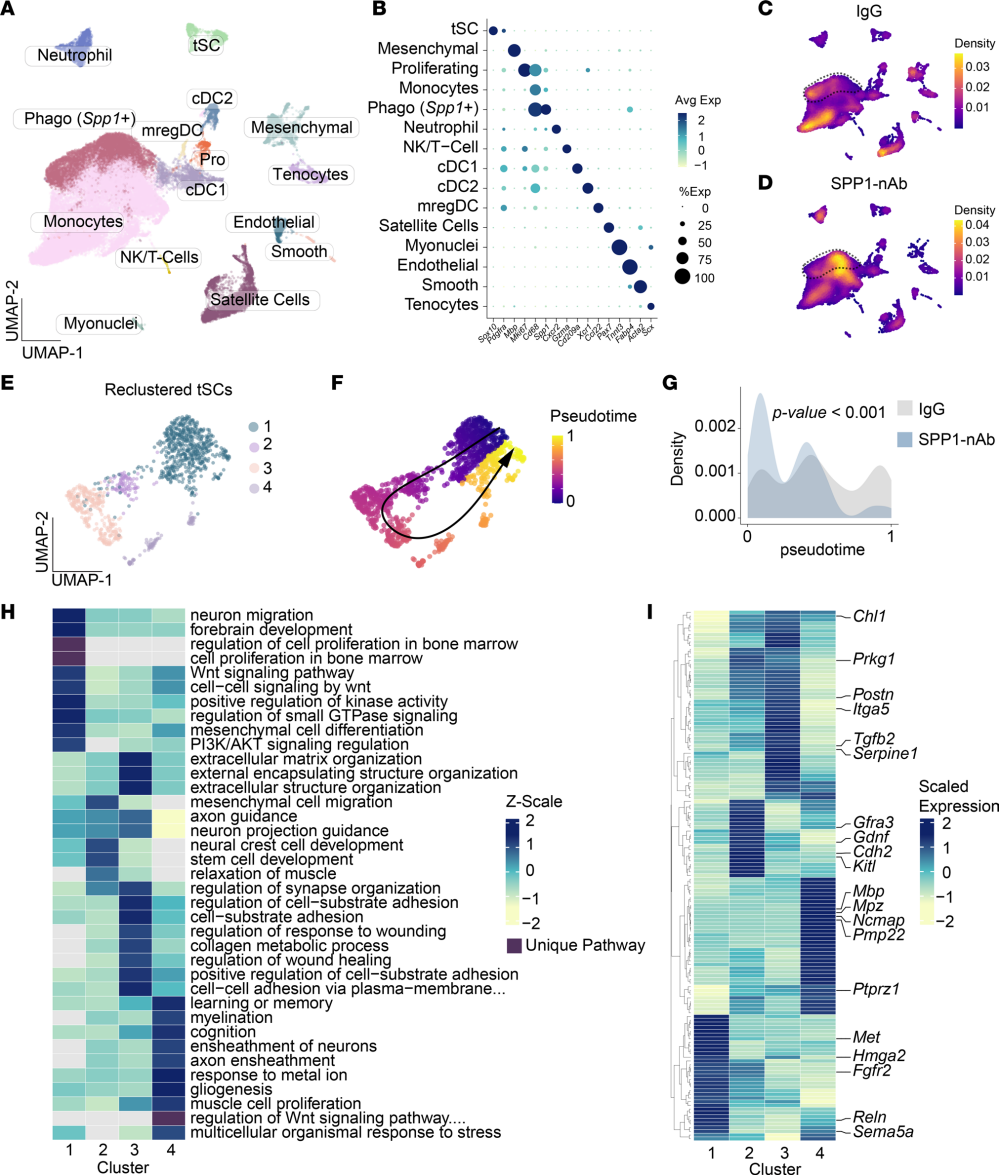
scRNA-Seq suggests that SPP1 neutralization stalls tSC state transitions after nerve injury. (**A**) UMAP of whole-muscle scRNA-Seq at 7 dpi (IgG vs. SPP1-nAb combined), annotated by major cell types, including terminal Schwann cells (tSCs) and an *Spp1^+^* phagocytic population. (**B**) Dot plot of canonical markers across annotated populations. (**C** and **D**) Density maps of IgG-treated (**C**) and SPP1-nAb–treated (**D**) cells projected onto the same manifold; dashed lines highlight the *Spp1^+^* cells. (**E**) Reclustering of the tSC subset identifies 4 transcriptional states (1 to 4). (**F**) Pseudotime ordering of tSCs with inferred trajectory (black arrow), indicating progression toward a late state and return toward a homeostatic node. (**G**) Pseudotime distributions by treatment reveal a significant left shift in SPP1-nAb–treated cells (2-tailed unpaired Student’s *t* test; *P* < 0.001), consistent with a stall in state progression. (**H**) GO pathway heatmap (*z* scored within term) across tSC states. Early Cluster 1: axon/neuron guidance and PI3K/AKT; Cluster 3: ECM organization/adhesion and TGF-β; Cluster 4: glial maturation/ensheathment; Cluster 2: neural-crest/motility. Purple ticks indicate pathways unique to a single state. (**I**) Aggregated, scaled expression heatmap (genes × clusters) for state-defining markers with callout labels.

To define how *Spp1* shapes the tSC response in vivo during denervation and reinnervation, we performed scRNA-Seq on TA muscles 7 days following peroneal nerve crush with administration of either IgG or SPP1-nAb. The atlas contained expected immune and stromal populations and a discrete tSC cluster (Figure 6, A and B). Density overlays on the common UMAP showed that, consistent with our scRNA-Seq analyses following nerve crush injury in untreated muscles (Figure 1H), *Spp1*^+^ phagocytic/myeloid cells persisted in both IgG- and SPP1-nAb–treated muscles. Interestingly, this cell population was enriched in SPP1-nAb–treated muscles compared with IgG controls (Figure 6, C and D), consistent with compensatory recruitment/retention of *Spp1*-expressing cells when extracellular SPP1 is neutralized. Compared with the uninjected dataset in Figure 1, both IgG and SPP1-nAb cohorts showed a clearer representation of conventional dendritic cell type 1 (cDC1), conventional DC type 2 (cDC2), and mature regulatory DCs (mregDCs) (Figure 6, A and B). This likely reflects contextual differences between assays, including sciatic versus peroneal injury and the repeated intramuscular injections that can recruit or mature DCs.

Focusing specifically on the tSCs, reclustering of tSCs yielded 4 transcriptional states (Figure 6E). Pseudotime analysis of the tSC clusters inferred a progression from an early injury/guidance state through an ECM/adhesion remodeling phase to a late glial maturation state, followed by a return toward a homeostatic node (Figure 6F). While tSCs from IgG-treated muscles traversed this loop, reaching the late maturation state and then turning back, cells from muscles in which SPP1 was neutralized accumulated earlier along the path, with a significant left-shift in pseudotime density (*P* < 0.001; Figure 6G), indicating stalled progression.

State-specific enrichment and aggregated expression (Figure 6, H and I) support a stepwise tSC program from Cluster 1 → 2 → 3 → 4. Cluster 1 showed neuron/axon guidance and PI3K/AKT signatures with transcripts including *Reln*, *Met*, *Sema5a*, *Fgfr2*, and *Hmga2*, consistent with tSCs directing reinnervation at NMJs and with growth/AKT modules linked to axon-glia interactions (24–29). Cluster 2 featured a motility/trophic crest–like program (*Kitl*, *Cdh2*, *Gdnf*, *Prkg1*, and *Gfra3*), in line with studies showing that trophic cues and cadherin signaling promote SC migration and neurite outgrowth during regeneration (30–32). Cluster 3 was enriched for ECM organization, adhesion, and TGF-β processes, marked by *Postn*, *Itga5*, *Serpine1*, *Tgfb2*, and *Chl1*; prior work shows that ECM-integrin pathways enable SC adhesion/migration after injury, periostin is induced in SCs and enhances remodeling, CHL1 guides regenerating motor axons, and TGF-β orchestrates injury-evoked SC states (17, 33–35). Cluster 4 expressed late glial/ensheathment genes (*Mpz*, *Pmp22*, *Mbp*, *Ncmap*, and *Ptprz1*), matching classic mSC markers (36–38).

To test the hypothesis that SPP1 neutralization affects migration and proliferation programs in tSCs, we performed a targeted pathway analysis on the IgG versus SPP1-nAb single-cell dataset. Within each tSC cluster, we computed differentially expressed genes, ran Gene Ontology (GO) Biological Process enrichment, and then selected terms annotated to migration or motility and to proliferation or cell cycle. We summarized enrichment as raw −log_10_(*P*)-adjusted values in cluster-by-term heatmaps (Supplemental Figure 7, A and B). Migration and motility terms were most prominent in Cluster 3, with additional neural-crest and glial migration terms in Cluster 2, consistent with sustained guidance and remodeling programs under SPP1 neutralization. In contrast, proliferation and cell cycle terms were concentrated in Cluster 4 and were minimal or absent in Cluster 2, and these proliferation signals were reduced in SPP1-nAb relative to IgG. Together with the left shift in pseudotime, this pattern indicates that lowering SPP1 activity in the muscle niche maintains tSCs in migration-rich states in Clusters 2–3, while blunting the late proliferative features that characterize Cluster 4 at the same postinjury time point.

Overall, these data suggest that SPP1 neutralization arrests tSC progression before the late glial program. Whereas IgG cells proceed from guidance to motility/trophic to ECM/adhesion and ultimately ensheathment, SPP1-nAb–treated cells accumulate in earlier states and exhibit diminished ECM/TGF-β and maturation modules, providing a mechanistic explanation for the reduced reinnervation observed in Figure 5.

## Discussion

To our knowledge, the current study boasts the most detailed scRNA-Seq repository to date of muscle-resident SCs, encompassing tSCs, across both healthy and denervated muscles. One prior study isolated tSCs using a dual mouse reporter that expressed both NG2-dsRed and S100GFP and performed bulk RNA-Seq (12, 39), but bulk sequencing does not allow for the distinction between different tSC subtypes or illuminate cell-specific responses to NMJ remodeling. The present results elucidate a previously unreported reactive population of tSCs that exhibits a transcriptional signature consistent with promoting synapse formation, enhancing cell adhesion, and organizing the ECM. In complementary scRNA-Seq experiments following SPP1 neutralization, we observed a treatment-dependent redistribution of tSC states after nerve injury. When SPP1 was reduced in the muscle niche, tSCs accumulated in early guidance and remodeling programs with sustained migration and ECM or adhesion signatures and showed diminished proliferation and ensheathment features. These patterns implicate SPP1 as both an early trigger and a permissive factor for state transitions. As a trigger, acute SPP1 availability initiates adhesion and motility signaling needed at the outset of the response, while as a permissive factor, persistent SPP1 enables tSCs to progress from guidance/remodeling into subsequent programs. When SPP1 is limited, initiation and progression are compromised, and cells remain in earlier states at the same time point.

Our data also demonstrate that tSCs are reactive, marked by increased proliferation underpinned by an intricate SPP1 signaling mechanism, bridging mSCs and tSCs. Our observations of marked elevations in both SPP1 gene expression and protein immunofluorescence in the acute phase after muscle denervation is consistent with prior reports of induction of SPP1 in samples from patients with nerve injury (23), and we show for the first time to our knowledge that SPP1 expression has functional relevance for the efficacy and/or efficiency of NMJ reinnervation. Our observations of increased tSC numbers following peroneal nerve injury that were notably decreased following intramuscular administration of an SPP1-nAb support the hypothesis that SPP1 is acting through tSCs to effect its integral role in NMJ regeneration. This conclusion is further supported by the diminished synaptic contact area, a pronounced percentage of denervated NMJs, and impaired recovery of functional innervation associated with reduced numbers of tSC in SPP1-nAb–treated muscles. The single-cell atlas clarifies the cellular sources and consequences of SPP1 signaling; at baseline and after spontaneous NMJ disruptions, mSCs contribute *Spp1*, whereas in the context of frank nerve injury, myeloid cells also supply SPP1, producing a niche in which both glial and immune compartments can deliver the ligand. Under SPP1 neutralization, we observed persistence or enrichment of *Spp1^+^* myeloid cells and a left shift in tSC pseudotime, together with attenuation of ECM/adhesion and late ensheathment modules. These findings are concordant with published mechanisms in which extracellular SPP1 binds CD44 or integrins to recruit FAK/Src and activate PI3K/AKT, thereby promoting adhesion, process extension, and survival in macrophages, fibroblasts, and glia. In those systems, interrupting SPP1 signaling diminishes motility and matrix engagement and reduces AKT-dependent growth or survival signals (40, 41). Our niche-level perturbation is consistent with that framework, in which lowering available SPP1 in the muscle environment likely reduced CD44/integrin/PI3K/AKT inputs onto tSCs and neighboring cells, which helps explain the stall in tSC state progression and the delayed structural and functional reinnervation we observe. We note that this experiment manipulated SPP1 within the niche without cell specificity, so indirect routes through SPP1-responsive immune or stromal cells may contribute to the tSC phenotype.

While upregulation of SPP1 in muscles of patients suffering from Duchenne muscular dystrophy (42) and in mice following exercise (43) has been reported, our study uncovers muscle-resident mSCs as the origin of this signaling, rather than or perhaps in addition to macrophages targeting fibro/adipogenic progenitors (FAPs) (43, 44). The strong induction of SPP1 signaling pathway transcripts and intense SPP1 immunofluorescence in skeletal muscle after nerve injury suggests SPP1 may have multiple functions to mediate muscle reinnervation. Integral to muscle reinnervation is Wallerian degeneration, a process governed by dynamic interactions among immune cells, FAPs, and SCs (45). Thus, it is reasonable to hypothesize that SPP1 engages with multiple cell types in response to severe nerve trauma to facilitate myelin clearance, ECM remodeling, and axon regeneration. Based on both imaging for protein and scRNA-Seq, our current findings implicate SPP1 signaling originating primarily from mSCs in the absence of frank nerve injury, with additional signaling emanating from SPP1^+^ phagocytic cells following traumatic denervating events. Furthermore, our findings of SPP1^+^ phagocytic cells, particularly following nerve crush, alongside the complete absence of classical mSCs, suggest a potential transition of SCs to a phagocytic phenotype during denervation. Consequently, the SPP1^+^ cells observed at 7 dpi may originate from both SCs and myeloid cells. This concept would integrate contradictory views in the field regarding whether myelin clearance is primarily driven by SCs or through hematogenous macrophages (46). While the current study does not address this question directly, there is clear evidence that SC-mediated phagocytosis is detected during the acute phase after injury, with hematogenous macrophages entering the injury site later (47, 48), and recent advancements in genetic mouse models and genomic technologies will allow further investigation to clarify the roles and origins of these SPP1^+^ cells in the context of varying types of muscle denervation.

SPP1 signaling between glial cells is further supported by recent investigations showing elevated levels of SPP1 in nerve samples after injury, which positively correlated with SC proliferation and survival (23). Furthermore, SPP1 has neuroprotective roles in the visual system and is expressed by reactive astrocytes to promote retinal ganglion cell survival after traumatic optic nerve damage (49). Beyond this regenerative aspect, SPP1 is implicated in pathological contexts, including tumor growth and metastasis, highlighting its versatility and likely the complexity of its regulation in promoting cell survival and growth in various contexts and cell types (50). For example, in pancreatic ductal adenocarcinoma, epithelial cells secrete SPP1 that binds integrin β3 (ITGB3, also known as CD61) on neighboring mesenchymal tumor cells and maintains a mesenchymal program. Moreover, genetic disruption of SPP1 or ITGB3 collapses this state and reduces metastasis, with accompanying changes in NF-κB, BMP2, and GREM1 signaling (51). This recent report indicating that SPP1 can signal through integrins to regulate cell state and invasion reinforces the notion that SPP1/integrin signaling is a potent, context-dependent regulator of adhesion and motility programs, consistent with our interpretation in injured muscle.

The synaptic regenerative signature we identified in tSCs during the response to denervation is distinctly characterized by the expression of *Gap43* and *Ntng1*. GAP43 is noted for its concentrated presence within regenerating growth cones of neurons and plays an indispensable role in axon guidance (52). The expression of GAP43 is not exclusive to neurons but is also found in reactive astrocytes and in tSCs after denervation, correlating with the elongation of tSC processes (53, 54). Beyond axonal guidance, GAP43 is also involved in the transfer of mitochondria between astrocytes and glioblastoma cells (55). Given that mitochondria are abundant within growth cones to meet energy demands (56), one might speculate that *Gap43*^+^ tSCs promote axon regeneration through mitochondrial transfer, thereby contributing to metabolic support. This proposition opens new areas for exploring unconventional pathways of axon regeneration and delineating the multifaceted roles of GAP43 in neural repair mechanisms. Netrins are also important for axon guidance and netrin-1 has been shown to promote SC migration and proliferation (57, 58). Recently, netrin-G1 was shown to facilitate signaling interactions between tSCs and sensory neurons, crucial for organogenesis in both hairy and nonhairy skin (59).

Cellular proliferation is universally recognized as a fundamental mechanism in tissue regeneration. Although the proliferation dynamics of mSCs have been extensively studied, the precise roles that SCs play during regeneration have not been established. A pertinent example is the marked 3- to 4-fold elevation in SC numbers observed within 1 week of sciatic nerve injury (60). While SC proliferation mediated by inhibition of cyclin D1 was ineffective in preventing nerve regeneration in the distal sciatic nerve stump (61), that study did not examine muscle-resident SCs or perform any functional assessment of NMJ transmission. tSCs not only proliferate after nerve injury but also extend cytoplasmic processes, promoting axonal sprouting, although the cellular signals remain understudied (2, 62). Further highlighting the importance of tSCs for nerve regeneration, a recent study that employed an anti-GD3 antibody to target the GD3 ganglioside on tSCs and trigger complement-mediated lysis and elimination showed reduced tSC numbers, decreased muscle reinnervation, and impaired functional recovery after a peroneal nerve injury (3). Our study expands on these previous studies by providing an understanding of the role of SPP1 signaling acting on SCs near the NMJ and its importance for the regeneration of NMJs. Our data following SPP1 neutralization further suggest that SPP1 supports not only tSC proliferation but also state transitions required for effective reinnervation.

Finally, our research further highlights the *Sod1*^–/–^ mouse as a useful model for understanding NMJ maintenance and repair processes. Surgical nerve crush injuries fully denervate every associated muscle fiber, leading to a lack of NMJ structural diversity. In contrast, young *Sod1*^–/–^ mice recapitulate what we postulate to be a more faithful representation of heterogeneous NMJ remodeling in the context of normal aging and/or degenerative neuromuscular diseases. In support of this contention are our observations during the remodeling phase of key characteristics reminiscent of NMJ regeneration, such as increased AChR area, tSC proliferation (2), polyinnervation (63), and axonal blebbing (64). Recognizing the potency of this model for investigating NMJ diversity and the response of muscle-resident SCs to denervation informed our decision to perform scRNA-Seq, which identified multiple subtypes of SCs, including what we believe to be a novel tSC subtype that our data collectively suggest is important and perhaps essential to NMJ regeneration. Overall, the current work deepens the existing knowledge on the intricate cellular processes of NMJ regeneration orchestrated by SCs, especially the importance of SPP1 signaling and distinct roles played by specialized SC subtypes. These insights both expand our understanding of neuromuscular biology and present promising avenues for targeted therapeutic strategies for debilitating neuromuscular disorders, including ALS and sarcopenia.

## Methods

### Sex as a biological variable.

Sex was balanced as evenly as possible across all experiments. An exception was the scRNA-Seq experiments, which used pooled male mice due to limited availability of female *S100GFP*-tg *Sod1*^–/–^ double mutants. In addition, this study was not powered to detect sex-specific effects, so male and female data were not analyzed separately for sex differences.

### Animal models.

All mice were bred at the University of Michigan, with *Sod1*^–/–^ and WT mice initially provided by Holly Van Remmen at the Oklahoma Medical Research Foundation (Oklahoma City, Oklahoma, USA). Mice expressing enhanced GFP driven by the S100B promoter to visualize SCs (*S100GFP*-tg Kosmos mice) (65) were provided by Alison Snyder-Warwick (when she was as at Washington University School of Medicine, St. Louis, Missouri, USA). *S100GFP*-tg mice were crossed with *Sod1*^–/–^ mice to generate mice containing a knockout allele for SOD1 and WT allele for the *Sod1* gene and the *S100GFP* transgene, *S100GFP*-tg *Sod1^+/–^*, which were crossed to produce a second generation of *Sod1*^–/–^ mice expressing GFP under the S100B promoter. To maintain a stable colony of *S100GFP*-tg *Sod1*^–/–^ mice, male *S100GFP*-tg *Sod1*^–/–^ and female S100GFP-tg *Sod1^+/–^* mice were bred consistently. Control mice in this study contain the genotype *S100GFP*-tg *Sod1*^+/+^, referred to as *S100GFP*-tg. Animals were sacrificed via intraperitoneal injection of tribromoethanol, followed by administration of a pneumothorax.

### In situ force testing.

Mice were anesthetized with initial intraperitoneal injections of tribromoethanol (250 mg/kg) with supplemental injections to maintain an adequate level of anesthesia during all procedures. GTN or TA muscle contractile properties were measured in situ, as described previously (66). Briefly, the whole muscle and associated nerve were isolated from surrounding tissues, and 4-0 silk suture was tied around the distal tendon, which was severed. The animal was placed on a platform warmed to maintain body temperature at 37°C. The hind limb was secured to the platform, and the tendon was tied to the lever arm of a servomotor (model 6650LR, Cambridge Technology). A continual drip of saline warmed to 37°C maintained muscle temperature. Muscles were activated either by stimulation of sciatic (GTN) or common peroneal (TA) nerve using a bipolar platinum wire electrode or by direct stimulation of the muscle via cuff electrodes wrapped around its proximal and distal ends. The voltage of 0.2-ms stimulus pulses and optimal muscle length (Lo) were adjusted to give maximum twitch force. At Lo, 300-ms trains of pulses were administered, increasing stimulation frequency until maximum isometric force (Po) was achieved. Muscle fiber length (Lf) was calculated based on Lf-to-Lo ratios of 0.45 for GTN muscles and 0.6 for TA muscles. Physiological cross-sectional area (CSA) was determined by dividing muscle mass by the product of Lf and 1.06 g/cm^3^, the density of mammalian skeletal muscle (67). Po was normalized by the CSA to calculate specific Po (sPo). Functional innervation was assessed by calculating the ratio of force production in response to nerve stimulation relative to the force elicited by direct muscle stimulation. A value of less than 1.0 indicates the fraction of muscle fibers that are unable to respond to nerve stimulation.

### Immunofluorescent staining and imaging.

Muscles were fixed in 4% paraformaldehyde (PFA) in PBS for 15 minutes and sucrose protected overnight with 20% sucrose, followed by embedding in optimal cutting temperature (OCT) compound (Tissue Tek) and stored at –80°C. For visualization of NMJ structure, muscles were dissected into bundles of 10–20 muscle fibers. Samples were treated with ice-cold methanol for 30 seconds, washed 3 times in PBS for 5 minutes each, and then blocked for 30 minutes at room temperature in 5% goat serum, PBS, Triton X-100 (0.5%), and 2% bovine serum albumin (BSA). Fab fragments (10% in blocking solution) were used to block endogenous IgG before applying primary antibodies. Stained muscle fiber bundles were mounted on slides and coverslips applied.

Primary antibodies used were as follows: a combination of NF (2H3) and SV2 (SV2) (Developmental Studies Hybridoma Bank) for labeling α-motor axon terminals and axons, anti-S100 (Dako, Z0311) to enhance labeling of all SCs, anti-Ki67 (rat) (ThermoFisher, 14-5698-82), anti-SPP1 (Abcam, ab8448), and anti-CD44 (Thermo Fisher Scientific, 50-143-16). Secondary antibodies consisted of Alexa Fluors that were conjugated to secondary antibodies (Life Technologies). α-Bungarotoxin (BTX) conjugated to Alexa Fluor 555 (Invitrogen, B35451) labeled the AChRs, and 4′,6-diamidino-2-phenylindole (DAPI) labeled nuclei.

A Nikon A1 high-sensitivity confocal laser scanning microscope with 40× oil immersion objective was used to capture 16-bit, 512 × 512-pixel frame size, *Z*-stack images with 1 μm interval. A minimum of 20–30 NMJs were acquired per animal.

### NMJ feature quantification.

Fiji ImageJ (NIH) was used to quantify NMJ features from confocal micrographs. Analyses were performed on maximum intensity projections in accordance with the NMJ-Morph image processing pipeline (21). Images were imported into ImageJ and cropped to isolate single en face NMJs, and only en face NMJs were included for quantification. Thresholding was used to generate binary masks for each NMJ component, which were used to extract morphometric measurements including area, perimeter, endplate area, and diameter (Supplemental Figure 4 and Supplemental Figure 4A). Overlap and tSC coverage measurements were quantified following NMJ-Morph guidelines (Supplemental Figure 4, D and E). Axon terminal blebs and polyinnervation events (defined as at least 2 axons converging on a single NMJ) were also scored, and blebs were recorded per NMJ (Supplemental Figure 4F).

### Quantification of perisynaptic and extrasynaptic Ki67^+^ nuclei.

Muscle fiber bundles from 2-month-old *S100GFP*-tg and *S100GFP*-tg *Sod1*^–/–^ were stained with anti-Ki67, DAPI, BTX, and anti-S100B to delineate cellular structures. Confocal imaging of fields containing NMJs along with surrounding areas was performed using a Nikon A1 microscope at ×20 and ×40 magnification. Perisynaptic Ki67^+^ nuclei were defined as DAPI^+^Ki67^+^ and overlapping with BTX staining. Extrasynaptic Ki67^+^ nuclei were those located within 50 μm from the nearest endplate. The quantification was normalized by the number of NMJs per image field to ensure comparability across fields and conditions. A minimum of 50 NMJs per muscle were analyzed.

### Single-cell isolation via FACS.

For tissue collection, mice were anesthetized with 3% isoflurane, then euthanized by cervical dislocation, bilateral pneumothorax, and removal of the heart. Hind limb muscles (TA and GTN) were quickly harvested using sterile surgical tools and placed in plastic petri dishes containing cold PBS. Using surgical scissors, muscles were minced and transferred into 50 mL conical tubes containing 20 mL of digest solution (2.5 U/mL Dispase II [Sigma] and 0.2% [~1000 U/mL] collagenase type II [Gibco], 1 mg/mL of hyaluronidase [Sigma] in Dulbecco’s modified Eagle medium [DMEM] [Gibco] per mouse). Samples were incubated on a rocker placed in a 37°C incubator for 60 minutes with manual pipetting every 30 minutes using an FBS-coated 10 mL serological pipette to break up tissue. Once the digestion was completed, 20 mL of F12 (Gibco Thermo Fisher) media containing 20% heat-inactivated FBS (Corning) was added to inactivate enzyme activity, and the solution was filtered through a 70-μm cell strainer into a new 50 mL conical tube and centrifuged at 300*g* for 5 minutes. Live cells were sorted from the suspension via addition of 1 μg of PI into each sample, which were filtered through 70-μm cell strainers before the FACS. Cell sorting was done using a FACSAria III cell sorter (BD Biosciences) and Discovery S8 cell sorter (BD Biosciences). GFP^+^PI^–^ and GFP^–^PI^–^ cells were sorted into 0.02% BSA/PBS solution for immediate processing.

### scRNA-Seq.

Freshly isolated single cells were sorted into staining solution, enumerated by hemocytometer, and resuspended in PBS. Cells were loaded into the 10X Genomics chromium single-cell controller for each sample and captured into nanoliter-scale gel bead-in-emulsions. cDNAs were prepared using the single cell protocol as per the manufacturer’s instructions and sequenced on a NovaSeq instrument (Illumina) with 26 bases for read 1 and 159 bases for read 2.

### scRNA-Seq data processing and analysis.

CellRanger 8.0.0 (10X Genomics) was used to process raw data using the GRCm39 reference with EGFP. The CellRanger workflow aligned sequencing reads to the GRCm38 transcriptome using the STAR aligner and exports count data (63). The CellRanger count command was run with default parameters. Filtered feature barcode data were imported into Seurat (v5) (68). Ambient RNA was removed using Decontx (69) and only cells with greater than 200 genes and less than 10% mitochondrial reads were included in our analysis. Cell doublets were removed using DoubletFinder (70). Scaling, normalization, variable gene selection, dimensionality reduction, and clustering were performed with default settings using the Seurat. Batch integration across all samples was performed using Harmony (71), and cell clustering was performed using a resolution equal to 1. Cell types were assigned to each cluster using known marker genes. Pathway analysis was performed using enrichGO() function in clusterProfiler (72) with the default parameters and used gene markers that were upregulated (log_2_FC > 1) in the tSC clusters compared to all other muscle-resident SCs. Upregulated genes were determined using the FindMarkers() function in Seurat. For each muscle-resident SC cluster, the top 10 biological processes (based on adjusted *P* values) were aggregated into a single data frame, retaining unique pathways only. Pathways were scaled as *z* scores across the dataset to compare pathway enrichments across muscle-resident SCs. A diffusion map was generated with the Destiny package (73), selecting the first 2 diffusion components for detailed mapping, and Slingshot (18) was used for trajectory analysis with the omega parameter set to “TRUE” to allow for multiple lineage origins. Cell-cell interaction analysis was performed with CellChat, focusing on “Secreted Signaling” pathways to assess intercellular communication within the muscle-resident SC niche.

### Whole-tissue RNA extraction and RT-qPCR analysis.

GTN and TA muscle samples were homogenized in TRIzol reagent (Invitrogen, Thermo Fisher Scientific) using a bead mill. RNA was isolated by phenol/chloroform extraction followed by isopropanol precipitation according to the manufacturer’s protocol and RNA yield was determined using a NanoDrop 2000c spectrophotometer (Thermo Fisher Scientific). Genomic DNA was removed by incubation with DNase I (Ambion, Thermo Fisher Scientific, AM2222) followed by its heat inactivation. Total RNA (1 μg) was reverse-transcribed to cDNA using SuperScript III Master Mix, Random Hexamers, and dNTPs (Invitrogen, Thermo Fisher Scientific) and RT-qPCR performed on a CFX96 Real-Time PCR Detection System (Bio-Rad, 1855195) in triplicate 20 μL reactions of iTaq Universal SYBR Green Supermix (Bio-Rad, 1725124) with 1 μM forward and reverse primer. Relative mRNA expression was determined using the 2^–ΔΔCt^ method with *Gapdh* serving as a control.

### Nerve injuries and neutralization of intramuscular SPP1.

Mice (10–16 weeks old) were subjected to bilateral sciatic or peroneal nerve crush to induce muscle denervation in both legs. *S100GFP*-tg mice were used for the antibody neutralization experiments, whereas WT C57BL/6J mice were used for the sciatic nerve crush experiments. Fine forceps (no. 5) were used to perform each nerve crush. For sciatic nerve injuries, tissues were collected at 0, 7, 14, or 28 dpi. For peroneal nerve injuries, a 10 μL intramuscular injection of SPP1-neutralizing antibody (2 μg; AF808, R&D Systems) was administered into the TA muscle using a Hamilton syringe and repeated every 2 days following the initial injury. Control mice received goat anti–mouse IgG (2 μg; 115-005-003, Jackson ImmunoResearch) on the same schedule. At 7 dpi, mice were anesthetized, and TA muscles were assessed for contractile function and collected for NMJ imaging.

### Statistics.

Data are presented as the mean ± SEM. All statistical analyses were performed using Prism 8 (GraphPad Software) and R version 4.4.1 (https://cran.r-project.org/bin/windows/base/old/4.4.1/). Between-group differences were tested by 2-tailed unpaired Student’s *t* tests (2 groups) or by a 1-way analysis of variance (ANOVA) followed by Dunnett’s multiple-comparison test. A 2-way ANOVA followed by Tukey’s multiple-comparison test was executed when 2 factors were involved. Differences were considered to be statistically significant at *P* less than 0.05.

### Data availability.

All raw and processed sequencing related data are available in the NCBI’s Gene Expression Omnibus (GEO GSE246865, GSE291457, and GSE311353). Values for all data points in graphs are reported in the Supporting Data Values file.

### Code availability.

All source code is available on GitHub: https://github.com/sdguzman/Muscle_SC

### Study approval.

All animal experiments were performed according to the NIH *Guide for the Care and Use of Laboratory Animals* (National Academies Press, 2011) and were approved by the University of Michigan Institutional Animal Care and Use Committee (IACUC) (PRO00008744).

## Author contributions

SDG, SVB, and PCDM conceived the study. SDG designed the research, conducted experiments, acquired and analyzed the data, and wrote the initial draft of the manuscript. AAM, CSD, LPR, PCDM, and SDG performed experiments and contributed to data acquisition and curation. SVB and PCDM contributed to study design, data interpretation, and critical revision of the manuscript. All authors provided input on the writing and approved the final version of the manuscript.

## Funding support

This work is the result of NIH funding, in whole or in part, and is subject to the NIH Public Access Policy. Through acceptance of this federal funding, the NIH has been given a right to make the work publicly available in PubMed Central.

- NIH grants R01 AG086251 (to SVB), R01 AG050676, P01 AG051442 (to SVB), P30 AR069620 (to SVB), and T32 AG000114 (to SDG)
- The American Physiological Society Porter Fellowship (to SDG).

## Supplementary Material

Supplemental data

Supporting data values

## Version 1 12/23/2025

In-Press Preview

## Version 2 02/23/2026

Electronic publication
