# Supplementary material for: Decoding muscle-resident Schwann cell dynamics during neuromuscular junction remodeling
Source: JCI Insight. 2025 Dec 23;11(4):e195917. doi: 10.1172/jci.insight.195917 (PMC12956019; doi:10.1172/jci.insight.195917)
Supplement: Supplemental data [file jciinsight-11-195917-s101.pdf]

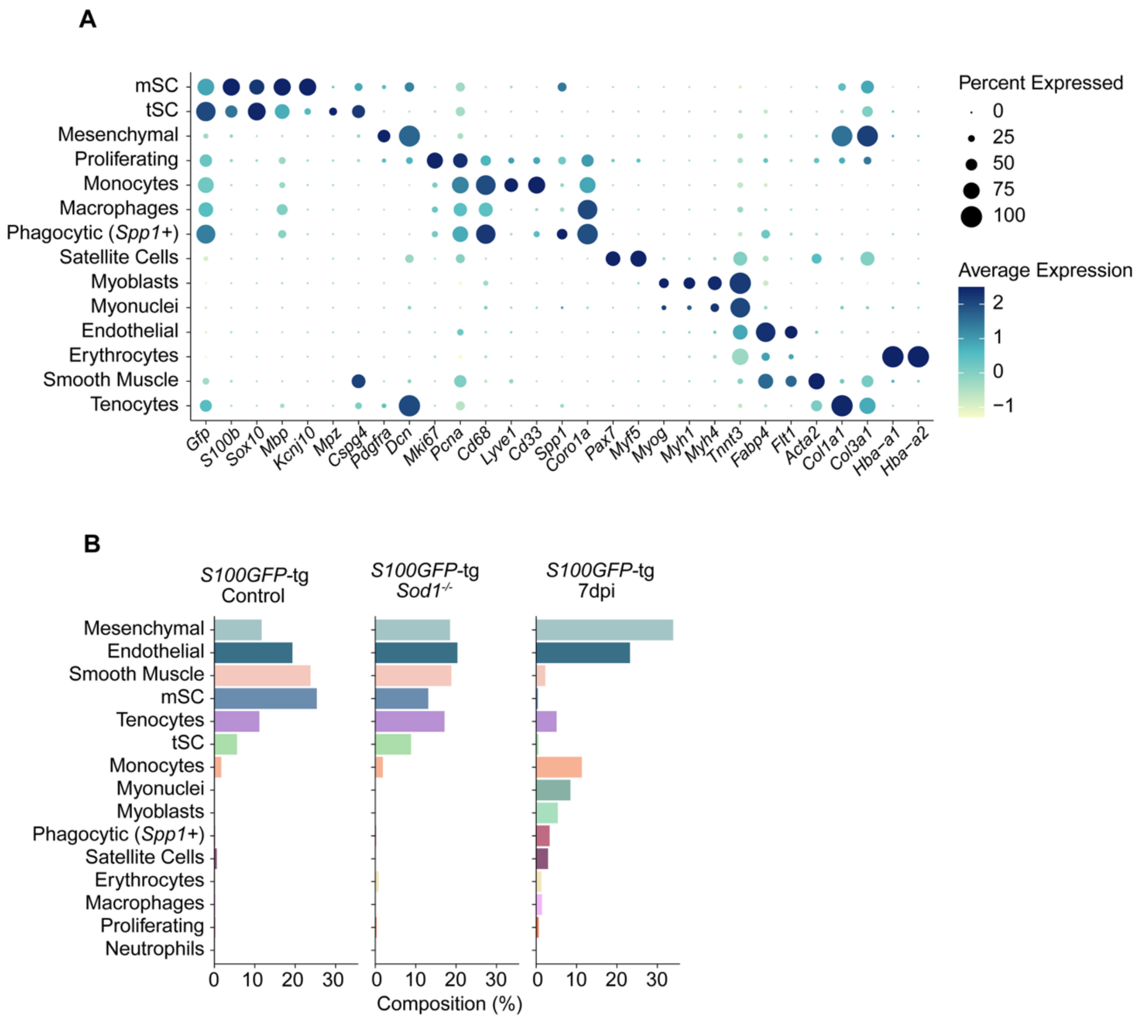

fig. S1. scRNA-Seq annotations and cell composition. (**A**) Dotplot showing the expression pattern of canonical markers, including *Gfp*, for each identified cell type cluster in the integrated dataset. (**B**) Bar plots showing the percentage (%) of each cell type cluster across all three groups.

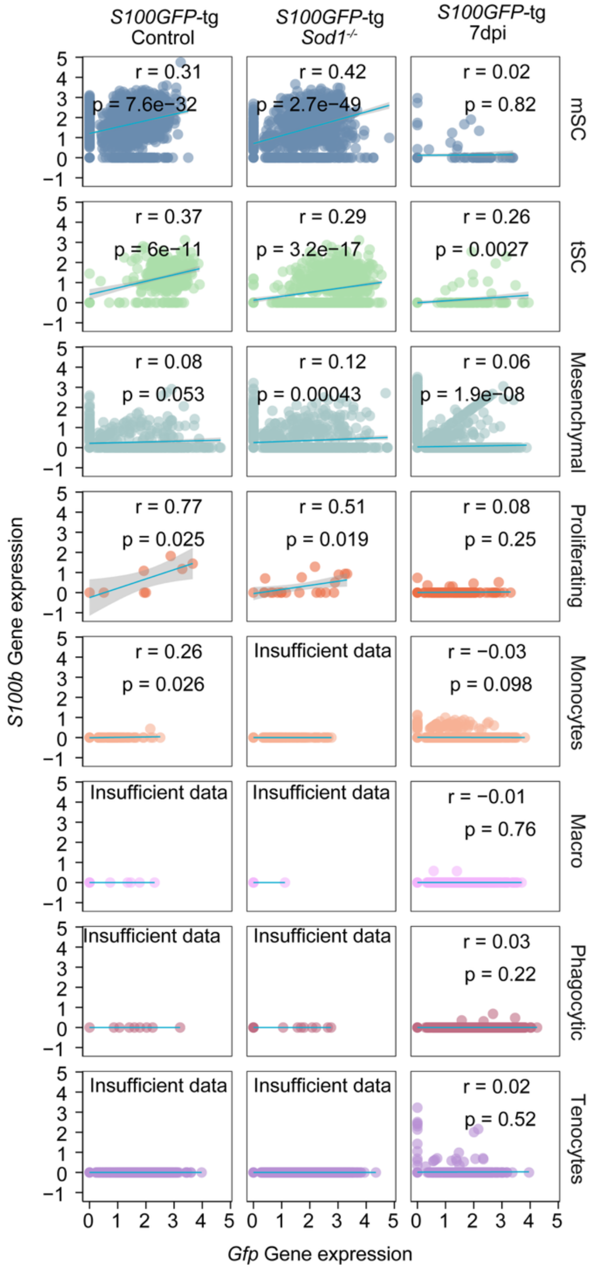

fig. S2. Correlation between Gfp and S100b expression across diverse cell types. Linear regression analysis of *Gfp* versus *S100b* expression in individual cells across various cell types within the integrated dataset that exhibited *Gfp* gene expression. Each subplot illustrates the regression line and includes the Pearson correlation coefficient, annotated where sufficient data points were available to perform the analysis.

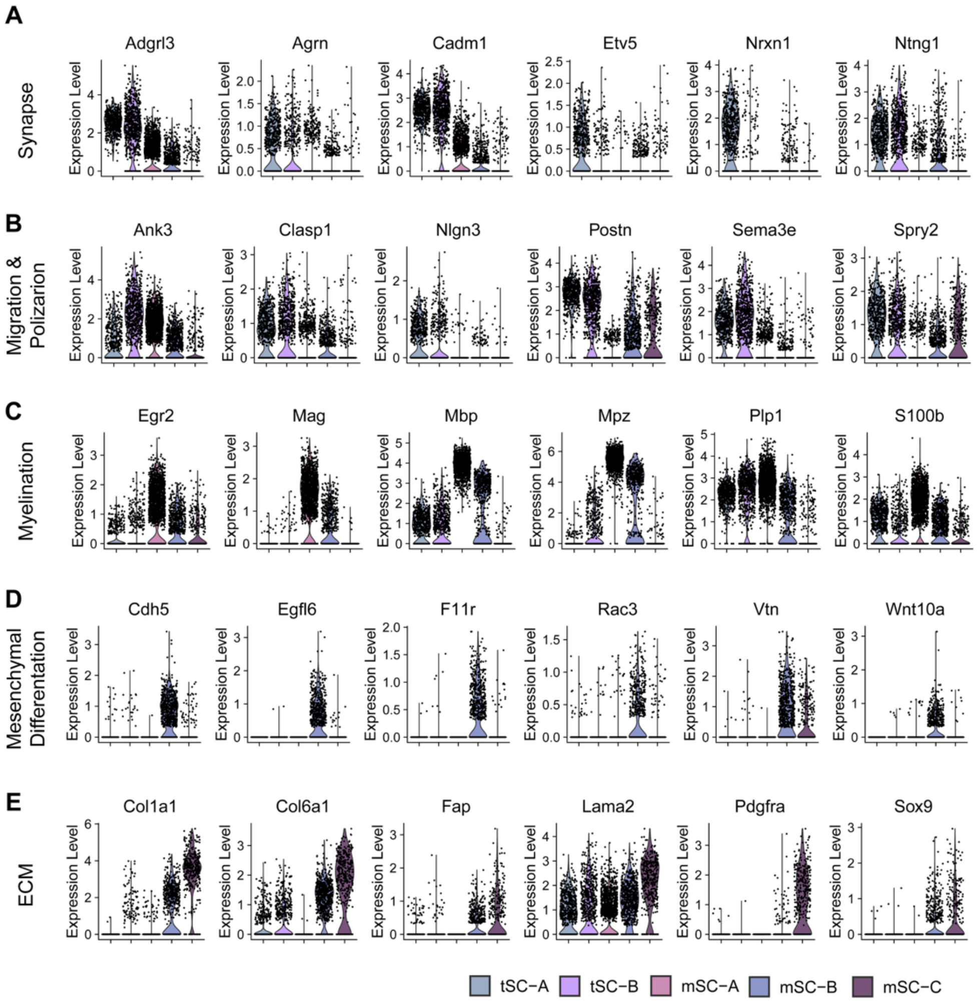

fig. S3. Differential expression of upregulated genes across pathway analysis categories in muscle-resident Schwann cells. Violin plots illustrate the expression patterns of enriched markers within the broad categories identified in Fig. 2—Synapse, Migration & Polarization, Myelination, Mesenchymal Differentiation, and ECM—across five muscle-resident Schwann cell subclusters.

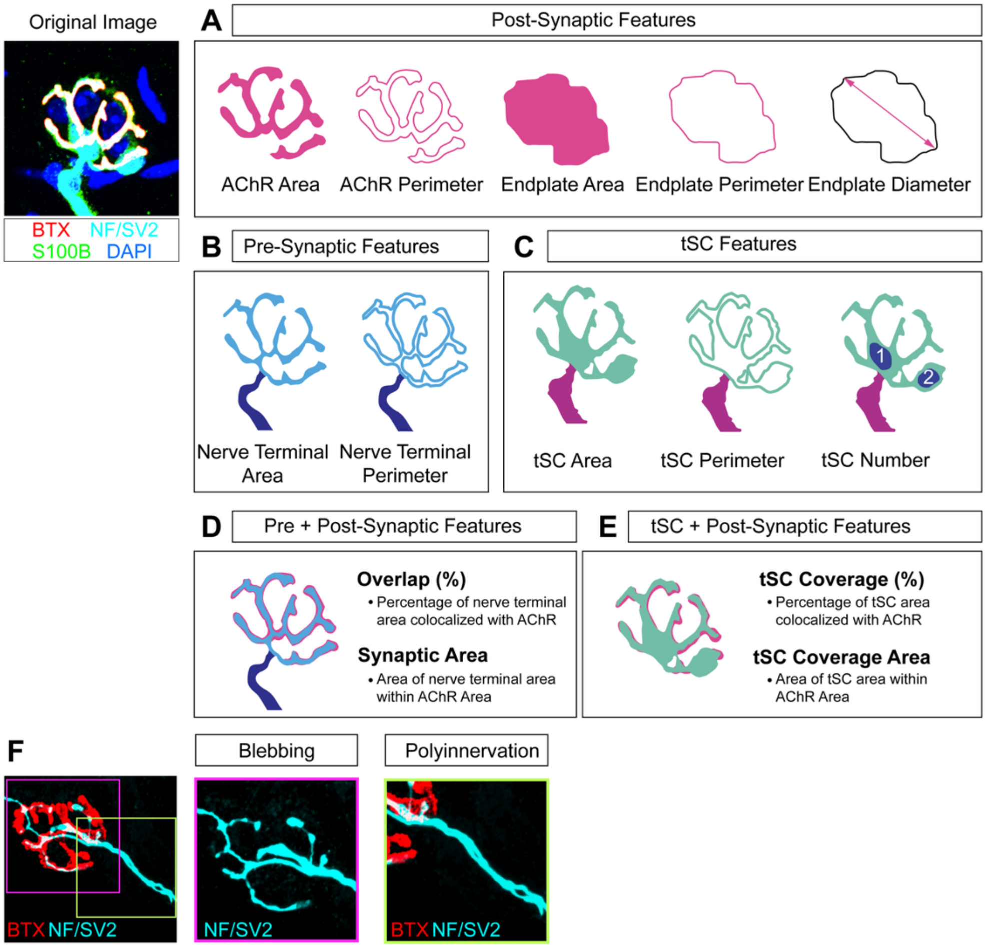

fig. S4. NMJ features analyzed. Binary masks were generated from the original image, and 16 en face NMJ morphological features were measured that represented the (**A**)**,** post-synaptic (AChR and Endplate), (**B**), pre-synaptic (nerve terminal, axonal blebbing, polyinnervation), and (**C**), tSC structures. These pixel measurements are visualized by distinct colorations in each mask: red for post-synaptic features, light blue for pre-synaptic features, and green for tSC structures. (**D**), In addition, the percentage "Overlap" between axon terminal and AChR areas were measured. This overlap was further quantified in terms of synaptic area (μm^2), denoting the area where the nerve terminal co-localizes with the AChR region. (**E**), Similarly, the "tSC coverage" was determined representing the tSC area that overlaps with the AChR region, and measured the "tSC Coverage Area," which is the expanse of tSC found within the AChR area. (**F**), The number of axon terminal blebs and and the presence of polyinnervation were also assessed for each NMJ.

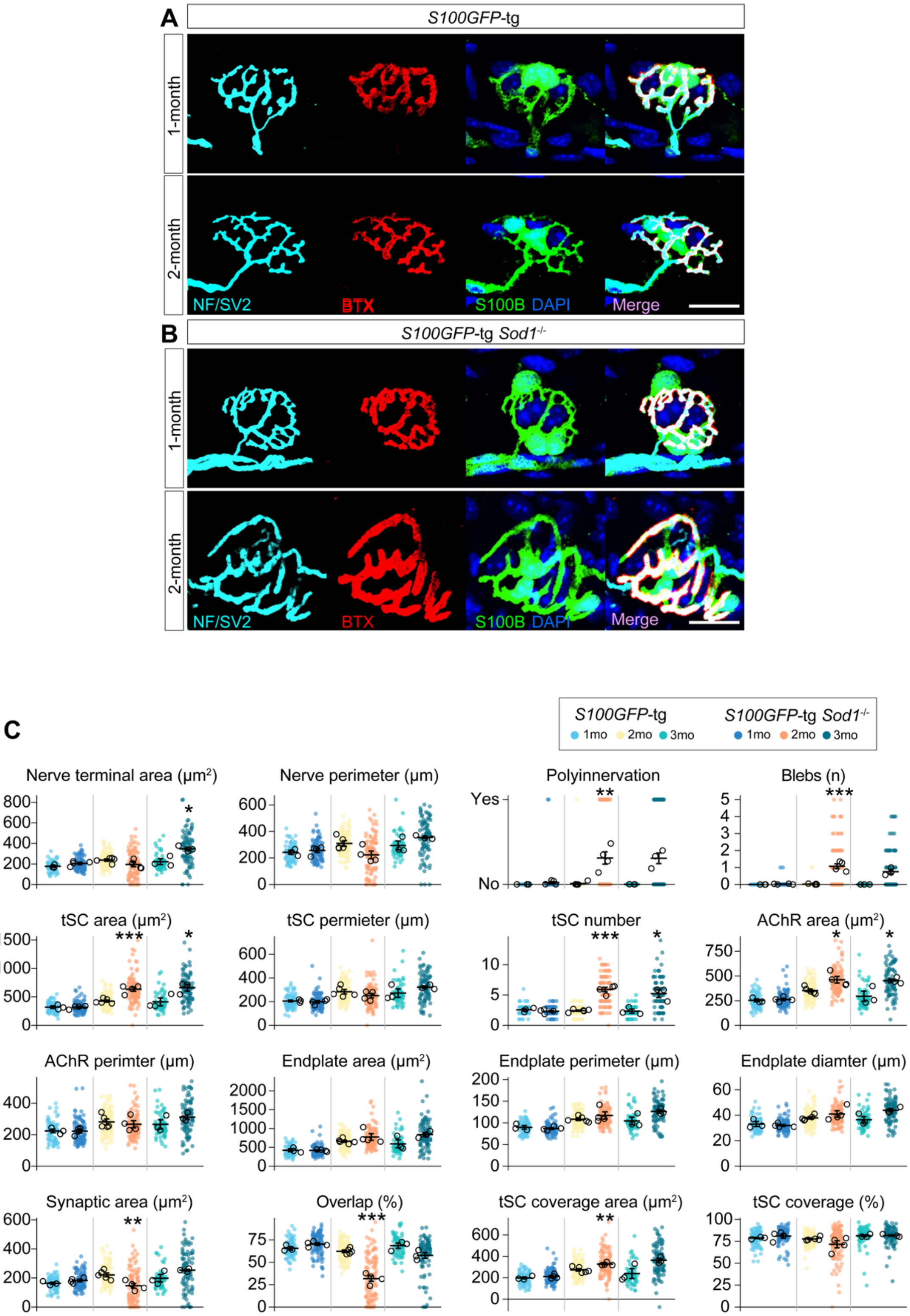

A
S100GFP-tg
1-month
2-month
NF/SV2
BTX
S100B DAPI
Merge
B
S100GFP-tg Sod1-/-
1-month
2-month
NF/SV2
BTX
S100B DAPI
Merge
C
S100GFP-tg
1mo 2mo 3mo
S100GFP-tg Sod1-/-
1mo 2mo 3mo
Nerve terminal area (μm2)
Nerve perimeter (μm)
Polyinnervation
Yes
No
Blebs (n)
tSC area (μm2)
tSC permieter (μm)
tSC number
AChR area (μm2)
AChR perimter (μm)
Endplate area (μm2)
Endplate perimeter (μm)
Endplate diamter (μm)
Synaptic area (μm2)
Overlap (%)
tSC coverage area (μm2)
tSC coverage (%)

fig. S5.| Analysis of NMJ in *S100GFP*-tg and *S100GFP*-tg *Sod1*$^{-/-}$ mice. (**A** and **B**) Images of representative NMJs from 1- and 3-month-old mice, stained for S100B (indicating Schwann cells in Green), NF/SV2 (Nerve in Cyan), bungarotoxin (AChR in Red), and DAPI (Nucleus in Blue). (**A**), Shows NMJs from *S100GFP*-tg mice, while (**B**) are NMJs from *S100GFP*-tg *Sod1*$^{-/-}$ mice. (**C**), Graphical representation of NMJ features in *S100GFP*-tg and *S100GFP*-tg *Sod1*$^{-/-}$ mice aged 1, 2, and 3 months. Each colored dot represents a single NMJ. Black circles denote the average for all NMJs in an individual animal. The horizontal bars show the overall mean for each group, while error bars illustrate the mean ± SEM. *Denotes $p < 0.05$ *S100GFP*-tg vs *S100GFP*-tg *Sod1*$^{-/-}$ by two tailed unpaired t-test. Scale bar equals 25 μm and images constrained to the same dimensions. **C**, N = 3-5/group.

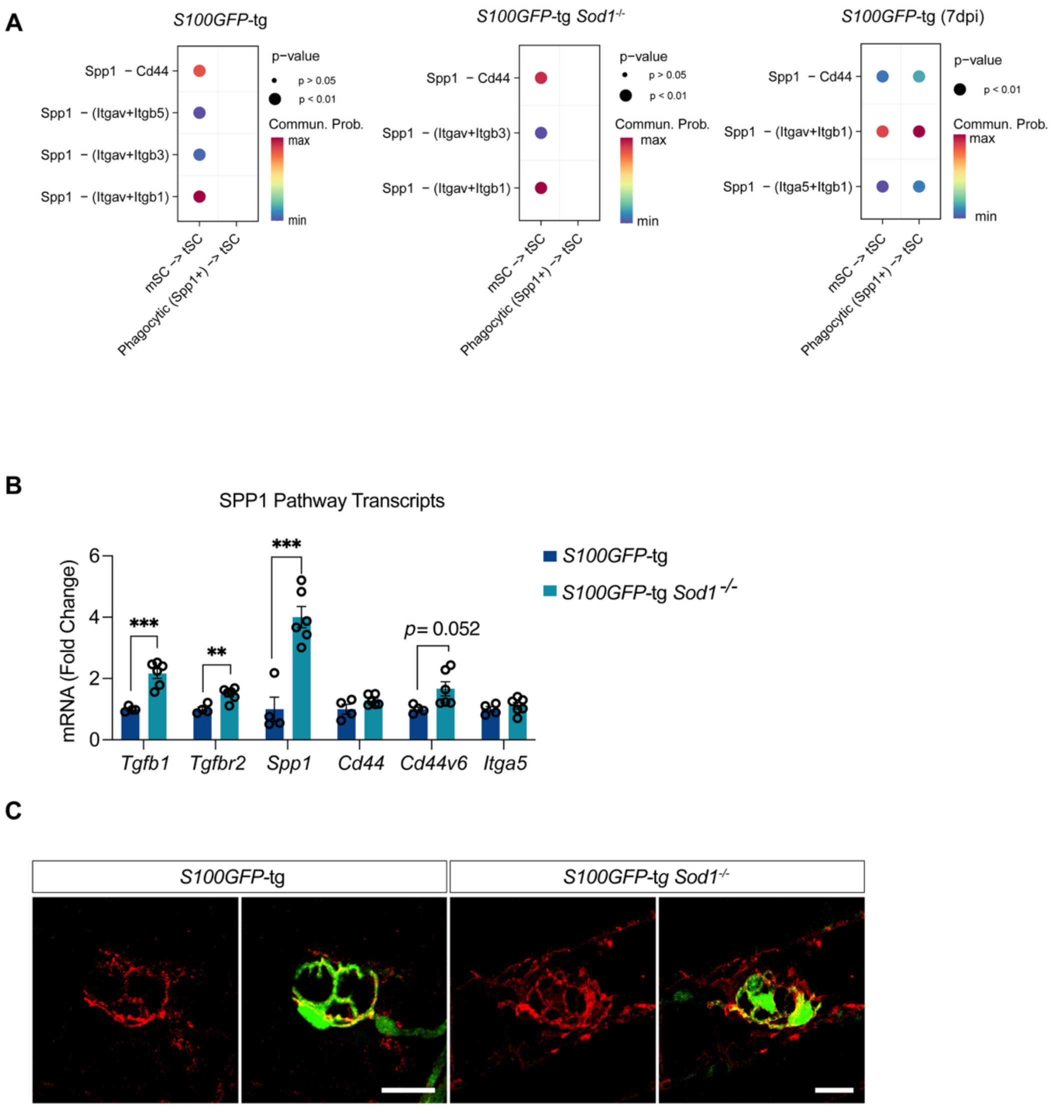
A
S100GFP-tg
S100GFP-tg Sod1-/-
S100GFP-tg (7dpi)
Spp1 – Cd44
Spp1 – (Itgav+Itgb5)
Spp1 – (Itgav+Itgb3)
Spp1 – (Itgav+Itgb1)
Spp1 – (Itga5+Itgb1)
p–value
p > 0.05
p < 0.01
Commun. Prob.
max
min
mSC -> tSC
Phagocytic (Spp1+) -> tSC
B
SPP1 Pathway Transcripts
mRNA (Fold Change)
S100GFP-tg
S100GFP-tg Sod1-/-
p= 0.052
Tgfb1
Tgfbr2
Spp1
Cd44
Cd44v6
Itga5
C
S100GFP-tg
S100GFP-tg Sod1-/-

fig S6. SPP1 signaling across different innervation states and confirmation of enhanced SPP1 gene expression and protein staining in *Sod1$^{-/-}$* mice muscles. (**A**) Dotplot visualizing signaling of the Spp1 ligand to Cd44 and integrin receptors, denoting interactions between mSCs and tSCs in both *S100GFP*-tg and *S100GFP*-tg *Sod1$^{-/-}$* , *S100GFP*-tg (7 dpi) mice. (**B**) mRNA levels determined by qPCR for several presumed components of the Spp1 signaling pathway for muscles of *S100GFP*-tg (n = 4) and *S100GFP*-tg *Sod1$^{-/-}$* (n = 6) mice. (**C**) Representative immunofluorescent images of NMJs stained for CD44 (Red) and S100B (Green). Open circles indicate values for individual mice and bars represent the mean across animals ± SEM. Scale bars represent 20 µm. *$p \leq 0.05$, **$p \leq 0.01$, ***$p \leq 0.001$, by two tailed unpaired t-test (*S100GFP*-tg vs *S100GFP*-tg *Sod1$^{-/-}$*).

A

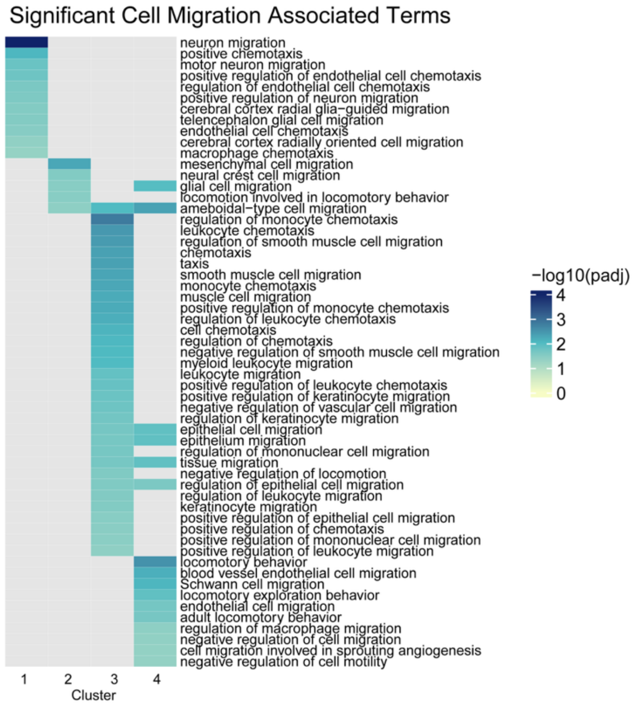
Significant Cell Migration Associated Terms
neuron migration
positive chemotaxis
motor neuron migration
positive regulation of endothelial cell chemotaxis
regulation of endothelial cell chemotaxis
positive regulation of neuron migration
cerebral cortex radial glia−guided migration
telencephalon glial cell migration
endothelial cell chemotaxis
cerebral cortex radially oriented cell migration
macrophage chemotaxis
mesenchymal cell migration
neural crest cell migration
glial cell migration
locomotion involved in locomotory behavior
ameboidal−type cell migration
regulation of monocyte chemotaxis
leukocyte chemotaxis
regulation of smooth muscle cell migration
chemotaxis
taxis
smooth muscle cell migration
monocyte chemotaxis
muscle cell migration
positive regulation of monocyte chemotaxis
regulation of leukocyte chemotaxis
cell chemotaxis
regulation of chemotaxis
negative regulation of smooth muscle cell migration
myeloid leukocyte migration
leukocyte migration
positive regulation of leukocyte chemotaxis
positive regulation of keratinocyte migration
negative regulation of vascular cell migration
regulation of keratinocyte migration
epithelial cell migration
epithelium migration
regulation of mononuclear cell migration
tissue migration
negative regulation of locomotion
regulation of epithelial cell migration
regulation of leukocyte migration
keratinocyte migration
positive regulation of epithelial cell migration
positive regulation of chemotaxis
positive regulation of mononuclear cell migration
positive regulation of leukocyte migration
locomotory behavior
blood vessel endothelial cell migration
Schwann cell migration
locomotory exploration behavior
endothelial cell migration
adult locomotory behavior
regulation of macrophage migration
negative regulation of cell migration
cell migration involved in sprouting angiogenesis
negative regulation of cell motility
1 2 3 4
Cluster
−log10(padj)
4
3
2
1
0

B

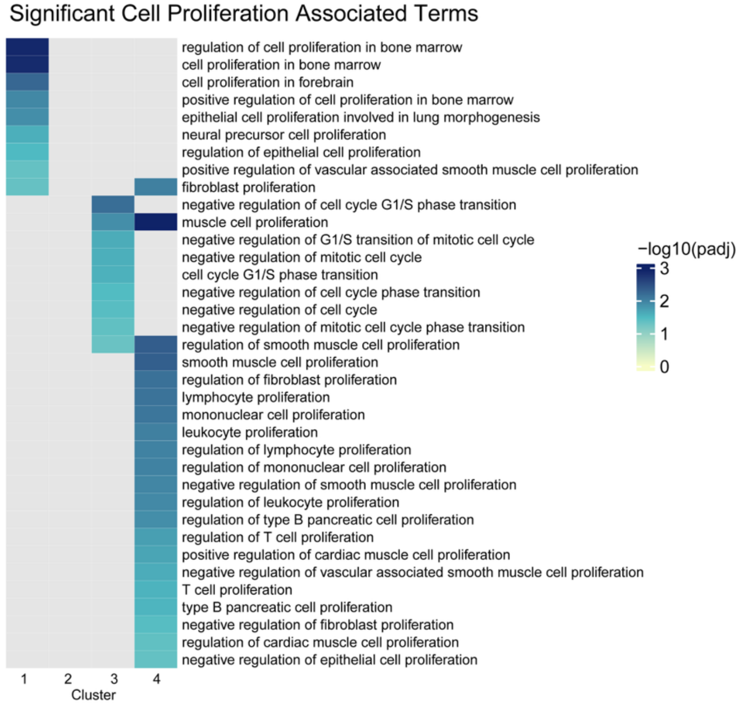
Significant Cell Proliferation Associated Terms
regulation of cell proliferation in bone marrow
cell proliferation in bone marrow
cell proliferation in forebrain
positive regulation of cell proliferation in bone marrow
epithelial cell proliferation involved in lung morphogenesis
neural precursor cell proliferation
regulation of epithelial cell proliferation
positive regulation of vascular associated smooth muscle cell proliferation
fibroblast proliferation
negative regulation of cell cycle G1/S phase transition
muscle cell proliferation
negative regulation of G1/S transition of mitotic cell cycle
negative regulation of mitotic cell cycle
cell cycle G1/S phase transition
negative regulation of cell cycle phase transition
negative regulation of cell cycle
negative regulation of mitotic cell cycle phase transition
regulation of smooth muscle cell proliferation
smooth muscle cell proliferation
regulation of fibroblast proliferation
lymphocyte proliferation
mononuclear cell proliferation
leukocyte proliferation
regulation of lymphocyte proliferation
regulation of mononuclear cell proliferation
negative regulation of smooth muscle cell proliferation
regulation of leukocyte proliferation
regulation of type B pancreatic cell proliferation
regulation of T cell proliferation
positive regulation of cardiac muscle cell proliferation
negative regulation of vascular associated smooth muscle cell proliferation
T cell proliferation
type B pancreatic cell proliferation
negative regulation of fibroblast proliferation
regulation of cardiac muscle cell proliferation
negative regulation of epithelial cell proliferation
1 2 3 4
Cluster
−log10(padj)
3
2
1
0

fig S7. SPP1 neutralization selectively impacts migration and proliferation programs in tSC clusters. (**A**) Heatmap of migration/motility–associated GO:Biological Process terms enriched within tSC Clusters 1–4. For each cluster, differentially expressed genes from IgG vs. SPP1-nAb were subjected to GO:BP enrichment; rows show curated migration/motility terms and cells display the raw −log10(p-adjusted) enrichment value for that cluster (higher values indicate stronger enrichment; gray denotes no significant term). Migration terms are most prominent in Cluster 3, with additional neural-crest/glial migration terms in Cluster 2. (**B**) Heatmap of proliferation/cell-cycle–associated GO:BP terms using the same analysis as in (**A**). Proliferation terms are largely confined to Cluster 4 and are minimal or absent in Cluster 2.
